# Shared *cis*-regulatory architecture identified across defense response genes is associated with broad-spectrum quantitative resistance in rice

**DOI:** 10.1038/s41598-018-38195-x

**Published:** 2019-02-07

**Authors:** Bradley W. Tonnessen, Ana M. Bossa-Castro, Ramil Mauleon, Nickolai Alexandrov, Jan E. Leach

**Affiliations:** 1 Colorado State University, Fort Collins, CO USA; 2 International Rice Research Institute, Manila, Philippines

## Abstract

Plant disease resistance that is durable and effective against diverse pathogens (broad-spectrum) is essential to stabilize crop production. Such resistance is frequently controlled by Quantitative Trait Loci (QTL), and often involves differential regulation of Defense Response (DR) genes. In this study, we sought to understand how expression of DR genes is orchestrated, with the long-term goal of enabling genome-wide breeding for more effective and durable resistance. We identified short sequence motifs in rice promoters that are shared across Broad-Spectrum DR (BS-DR) genes co-expressed after challenge with three major rice pathogens (*Magnaporthe oryzae*, *Rhizoctonia solani*, and *Xanthomonas oryzae* pv. *oryzae*) and several chemical elicitors. Specific groupings of these BS-DR-associated motifs, called *cis*-Regulatory Modules (CRMs), are enriched in DR gene promoters, and the CRMs include *cis*-elements known to be involved in disease resistance. Polymorphisms in CRMs occur in promoters of genes in resistant relative to susceptible BS-DR haplotypes providing evidence that these CRMs have a predictive role in the contribution of other BS-DR genes to resistance. Therefore, we predict that a CRM signature within BS-DR gene promoters can be used as a marker for future breeding practices to enrich for the most responsive and effective BS-DR genes across the genome.

## Introduction

Rice (*Oryza sativa L*.) is a staple food for more than 60% of humanity^1^. The growing human demand, together with losses caused by pathogens worldwide, require novel strategies and agricultural practices to increase global production of rice. Three major rice diseases, blast, sheath blight, and bacterial blight, can cause up to 100%, 25%, and 70% yield loss, respectively^2^. Identifying and understanding the genetic and molecular mechanisms of rice defense against the pathogens causing these diseases are important steps in the development of varieties with durable resistance.

Plant immunity consists of a two-tiered response system. Basal resistance enables plants to recognize conserved Microbe-Associated Molecular Patterns (MAMPs) through Pattern Recognition Receptors (PRRs), eliciting a downstream defense response (DR) known as Pattern-Triggered Immunity (PTI)^3^. Diverse metabolic functions work together during PTI, and the loci attributed to this response are known as DR genes. This DR gene-mediated process is also known as quantitative resistance due to its polygenic nature, and the ubiquity of the response is seen through resistance to diverse pathogen species, i.e. broad-spectrum^4^. Pathogens overcome PTI through the secretion of effectors, which inhibit aspects of the DR or increase host susceptibility, promoting a stronger infection. Plants, in turn, have developed resistance (*R*) proteins, that recognize the presence or activity of these effectors and activate a strong defense response called the Effector-Triggered Immunity (ETI)^3^. In crops such as rice, breeding for ETI in elite varieties is the main strategy for developing disease resistance. However, this single gene resistance is frequently unstable due to adaptation of the pathogen to circumvent the *R* gene mechanism^5^. The genotypic attributes that predict a strong PTI against a broad-spectrum of pathogens are far less characterized, but are key to breeding for adaptation and durability under disease pressure.

In our search for durable, broad-spectrum resistance, we focused on the many DR genes present in rice^6–10^. The activities of DR gene products are non-species specific, meaning they contribute to the broad-spectrum defense response (BS-DR)^11–13^. DR gene products function in the biosynthesis pathways of hormones such as Jasmonic Acid (JA), Salicylic Acid (SA), and Indole-3-acetic acid (IAA), Mitogen-Activated Protein Kinase (MAPK) signaling, thiamine biosynthesis, carbon metabolism, oxidative bursts, phenylpropanoid biosynthesis, and other diverse processes^14–19^. Quantitative Trait Loci (QTL) frequently contain DR genes or DR gene families as seen in rice, pepper, common bean, and wheat^6–10,20–22^. Each QTL imparts a partial contribution to resistance, and pyramiding multiple QTL to include many functional DR genes has proven a useful predictor of durable resistance in wheat and rice^6,12,23–25^. Thus, understanding how the resistant alleles of DR gene loci collaborate to improve the plant’s DR, and searching for those alleles across all potential DR genes, can provide insight into breeding for durable and broad-spectrum resistance throughout the genome.

In several cases, the functional difference between the resistant and susceptible DR gene haplotypes of a QTL are not in the coding region, but are due to polymorphisms in promoters. A promoter often contains small sequence motifs, classified as *cis*-elements, that recruit proteins or induce other regulatory mechanisms to modulate transcription. Mutations at these sites alter the DR gene’s functionality during pathogen infection. The germin-like protein, *OsGLP8-6*, is a key contributor among 12 *OsGLP8* family genes found on a QTL for rice blast resistance on chromosome 8^12^. This gene is classified as a BS-DR gene, as the expression of *OsGLP8-6* enhances resistance to the pathogens *M*. *oryzae*, *R*. *solani* and *X*. *oryzae* pv. *oryzae*^12,26^. An 856 bp promoter insertion that contains known defense-responsive *cis*-elements is present in the resistant haplotype of *OsGLP8-6*, and the gene shows faster and higher expression relative to the susceptible haplotype^26^. Another BS-DR gene, *OsOXO4*, is an oxalate oxidase and a member of a gene family of four in a QTL for resistance to *M*. *oryzae;* this gene also contributes to resistance to *R*. *solani*^27,28^. The *OsOXO4* promoter haplotype found in resistant lines contains an insertion (26 bp) with known defense *cis*-elements^27^. *OsGH3-2* is a rice IAA-amido synthetase BS-DR gene, located within a QTL for resistance against both *X*. *oryzae* pv. *oryzae* and *M*. *oryzae*, and contains promoter polymorphisms between resistant and susceptible alleles^14^. Promoter differences are also present between resistant and susceptible haplotypes in the CCCH-type zinc finger nucleic acid-binding protein, *OsC3H12*, which contributes to resistance to *X*. *oryzae* pv. *oryzae*^29^. In all these examples, the differences between resistance and susceptibility are polymorphisms that affect only transcription, not protein function, suggesting that the timing and intensity of BS-DR gene regulation are key to effective basal resistance. Identifying promoter elements associated with highly responsive BS-DR genes may provide genetic markers to facilitate accumulation of effective BS-DR genes.

The BS-DR genes described above are large effect genes, meaning the disease phenotype is noticeably altered if the gene is mutated or overexpressed. However, the intrinsic nature of BS-DR genes is quantitative, so many contributing genes will have a small effect that is difficult to detect. During the DR, the transcription of many genes is modulated, and the co-expression of BS-DR genes is critical for a well-orchestrated DR. Understanding regulation of BS-DR genes on a genomic scale, rather than restricted by QTL, can lead to a more comprehensive set of markers for broad-spectrum, quantitative resistance. The sequences of any one *cis*-element can be found in promoters throughout the genome, thus many resistant alleles of DR gene promoters may contain similar signatures. Here, we identify promoter motifs and promoter architectures that are shared across co-expressed BS-DR genes, and identify patterns that could predict effective BS-DR phenotypes and allow discovery of novel BS-DR genes throughout the genome. Using these sequence signatures for molecular marker-assisted breeding could ensure the preservation of a more durable and broad-spectrum resistance in the selected progeny.

## Results and Discussion

### Identifying co-expressed BS-DR genes across the rice genome

A defense response (DR) gene either promotes plant defense, or mediates other cellular processes to increase plant fitness against multiple diseases^11,21,30^. Thus, when considering the various infection mechanisms and host physiological responses to multiple and diverse pathogens, the DR exploits the plasticity of the plant’s entire genome. Given the pivotal role of broad-spectrum defense response (BS-DR) genes in modulating the pathways involved in resistance, a well-orchestrated transcriptional response is necessary^11^. Searching for signatures in co-expressed BS-DR genes will shed light on this subject, since co-expressed genes likely retain similar transcription regulatory elements. To this aim, we first identified a set of BS-DR genes from Nipponbare and IR64 rice genomes, representing two varietal groups widely used in rice breeding, Japonica and Indica, respectively.

Gene expression data from 44 published studies (Table S1, Fig. S1) were used to identify condition-dependent co-expressed gene clusters, with the condition consisting of both biotic and chemical DR elicitors. To find co-expressed BS-DR genes that respond to a broad-spectrum of diseases, the included studies measured transcriptomes of rice responses to rice blast (*M*. *oryzae*), sheath blight (*R*. *solani*) and bacterial blight (*X*. *oryzae* pv. *oryzae*), as well as the chemical defense elicitors benzothiadiazole (BTH), JA, cellulose, and chitin (Table S1). Combined co-expression clustering of 14,688 genes from the studies showed 65 clusters with average of 226 genes (Tables S2 and S3, Fig. S2). BS-DR gene clusters from these results were selected based on enrichment of both DR-related Gene Ontology (GO) terms (DR-GO terms) (Table S4) and DR genes functionally associated (FA) with plant defense (FA-DR genes) (Tables S5–S7). Seventeen of the 65 co-expressed clusters were enriched in DR-GO terms (Table S8).

**Figure 1:**
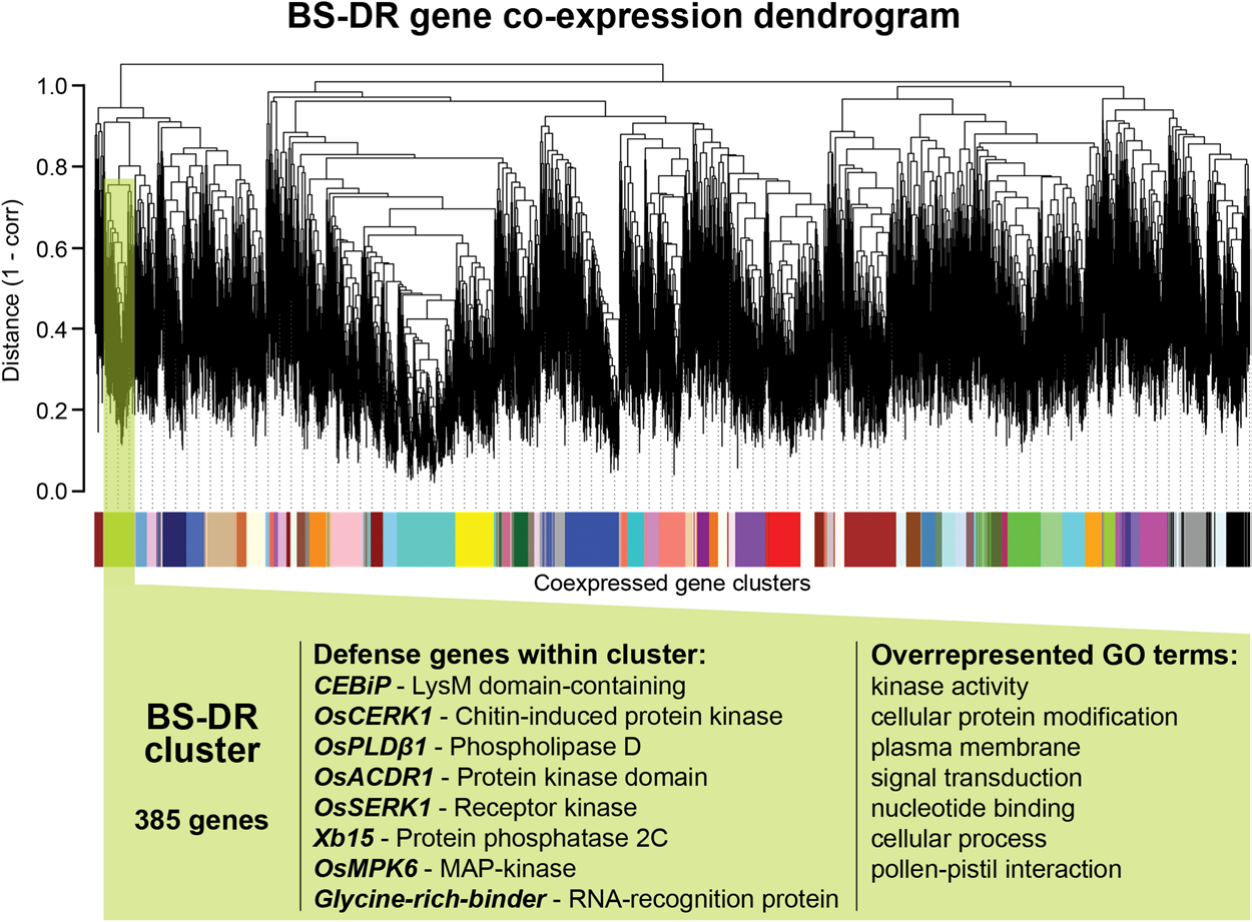
BS-DR gene co-expression dendrogram. Results of the average linkage hierarchical clustering of distance measures (1-PearsonCC). Colors along the base are distinct co-expression clusters (Fig. S2, Table S3). Highlighted in green is the BS-DR gene co-expression cluster. Known FA-DR genes listed are enriched in the highlighted cluster (Fisher Test BH correction, *P*-value: 0.0014, Tables S5 and S6). DR-related Gene Ontology terms that were significantly enriched in the highlighted cluster are given (Fisher BH correction FDR 0.05; Tables S4 and S7).

Only one cluster, containing 385 genes, deemed the “BS-DR cluster” (Table S9), was also enriched in FA-DR genes (Fig. 1, Table S6). Presence of genes within the BS-DR cluster suggests common regulation during DR, and therefore, we predicted their promoters might contain common sequence patterns. The following analysis focuses on identifying promoter elements associated with this single BS-DR cluster.

### Constructing an aligned “Promoterome” for two distinct rice varieties

Position of putative *cis-*elements in the promoter relative to the downstream gene is important for deducing their functionality^31^. A first step in identifying similar promoter motif architecture specific to BS-DR genes was to ensure correct alignment to the transcription start site (TSS) across the genome for accurate cross-comparison of promoters. The occurrences of each nucleotide for every promoter from the proposed TSS to 2 Kb upstream were counted. Nucleotide occurrences did not vary from −2 Kb to −500 bp, thus only the 100 bp region of the promoter is given (Fig. S3). The occurrence of C/G was consistently lower than A/T until −400 bp where C/G become the dominant nucleotides. Counts of A/T were relatively abundant within −35 to −24 of the TSS. This is the TATA box site, and it occurs in 24% of total genes in both rice varieties, a value comparable to the expected value of 19% in rice^32^. Cytosine was located right before the TSS, at position −1, in about 47% of promoters, and is indicative of transcribed genes^33^. These IR64 and Nipponbare promoteromes, appropriately aligned to the TSS, were used as two separate sequence sets in the position-specific motif searches described below.

### BS-DR gene-associated short sequence motifs have functionality in known defense pathways

**Figure 2:**
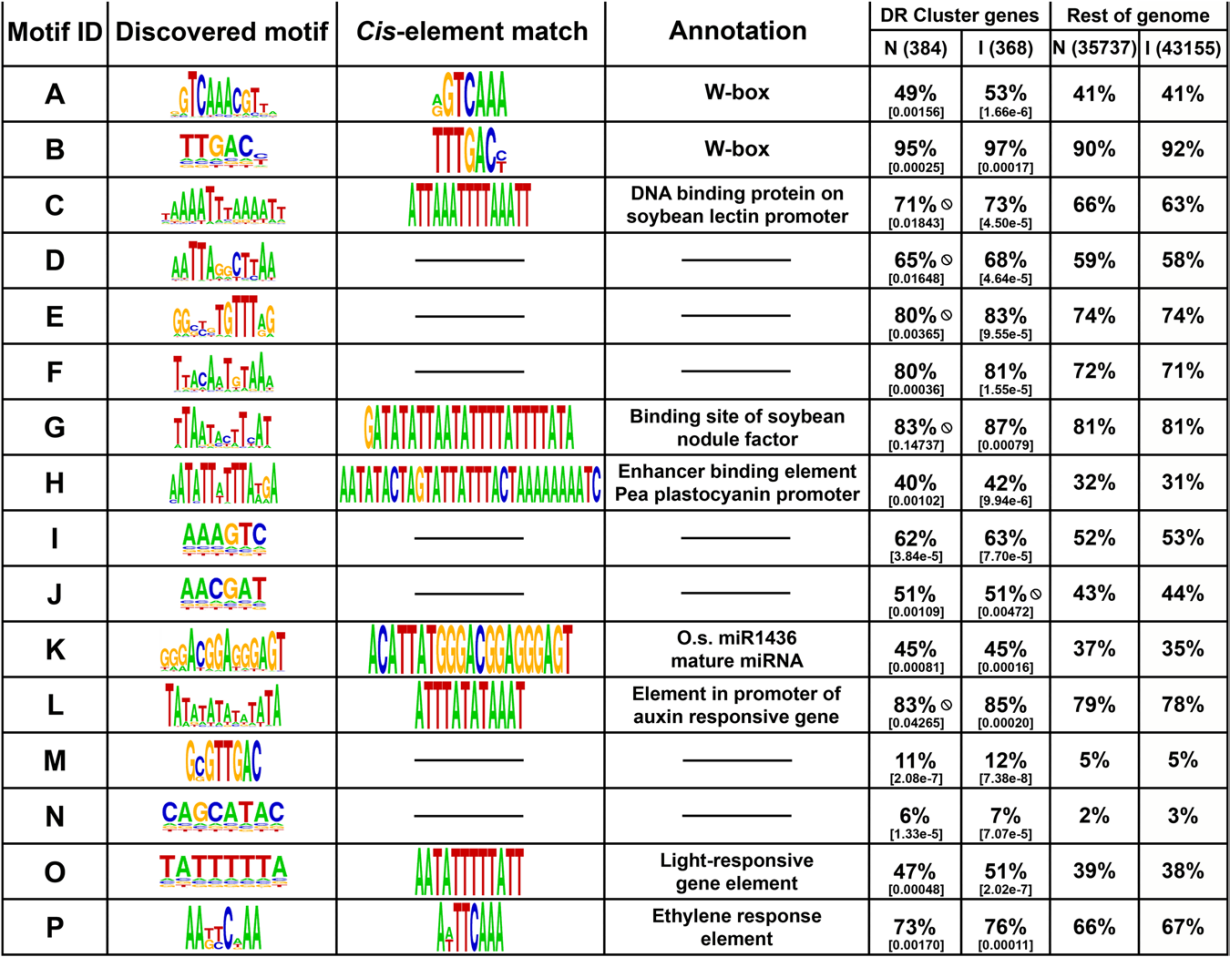
Short sequence motifs enriched in BS-DR gene promoters. The *de novo* discovered motif is given in the second column, and the third column shows the verified known *cis*-element (if any) found from public databases, followed by an annotation. The percent of gene promoters with the given motif is shown for both Nipponbare (N) and IR64 (I) in the BS-DR gene cluster or rest of the genome. Total number of genes for each set is given in parentheses next to the variety identifier. Cells that are followed by an interdictory circle were not found to be significant (*P*-values given in brackets, Fisher Exact Test, BH correction FDR 0.05).

To identify shared regulatory signatures of BS-DR co-expression cluster genes, we first detected overrepresented short sequence motifs in their respective promoters relative to the rest of the promoterome using the program Gimmemotifs^34^. Short sequence motifs, ranging from 6 to 15 bases, were detected in the promoters of genes in the co-expressed BS-DR cluster (Fig. 2). The top motifs found from each component algorithm were clustered by similarity, and merged using weighted information scores^34^. Enrichment was determined in the BS-DR cluster in promoteromes of both varieties (Bonferroni *P*-value < 0.00156) (Fig. 2). Only motifs enriched in at least one variety were included in further analysis.

Nine out of sixteen motifs found in this *de novo* method matched known *cis-*elements, all of which have a role in the plant-microbe interaction. Motifs A and B matched the core DNA binding element of the WRKY transcription factor; these *cis*-elements are essential for resistance to rice blast, sheath blight and bacterial blight^35–37^. Motif C matches a regulatory element on the promoters of lectins, which are inferred to be involved in resistance through protein-carbohydrate binding during the DR^38^. Motif G matches a nodule factor *cis-*element that coordinates communication and control of bacterial population between the host plant and *Rhizobium* bacteria^39^. The major form of auxin, IAA, negatively regulates defense through induction of expansin-mediated cell wall loosening^40^, and genes with promoters that harbor the auxin-responsive factor of Motif L may contribute positively or negatively to this susceptibility response. Motif P matches with an ethylene response element. Ethylene regulates host defense, and is found at higher levels in resistant interactions with *M*. *oryzae*^41^. A different type of motif aligned with a processed miRNA (Motif K), suggesting diverse roles of these BS-DR promoter elements.

These motifs are represented in many promoters across the genome, however, they are enriched in the promoters of the BS-DR gene cluster. Although small sequence motifs can be indicative of transcriptional control, *cis*-elements do not usually work alone. Multiple *cis*-elements often function together to regulate genes involved in both the DR and abiotic stress^31,42,43^. To better understand BS-DR gene regulation, we looked at the overall structure of promoters to determine if specific groupings of these multiple motifs were distinct to BS-DR promoters.

### BS-DR motifs are organized into cis-regulatory modules (CRMs) that predict diverse defense mechanisms

**Figure 3:**
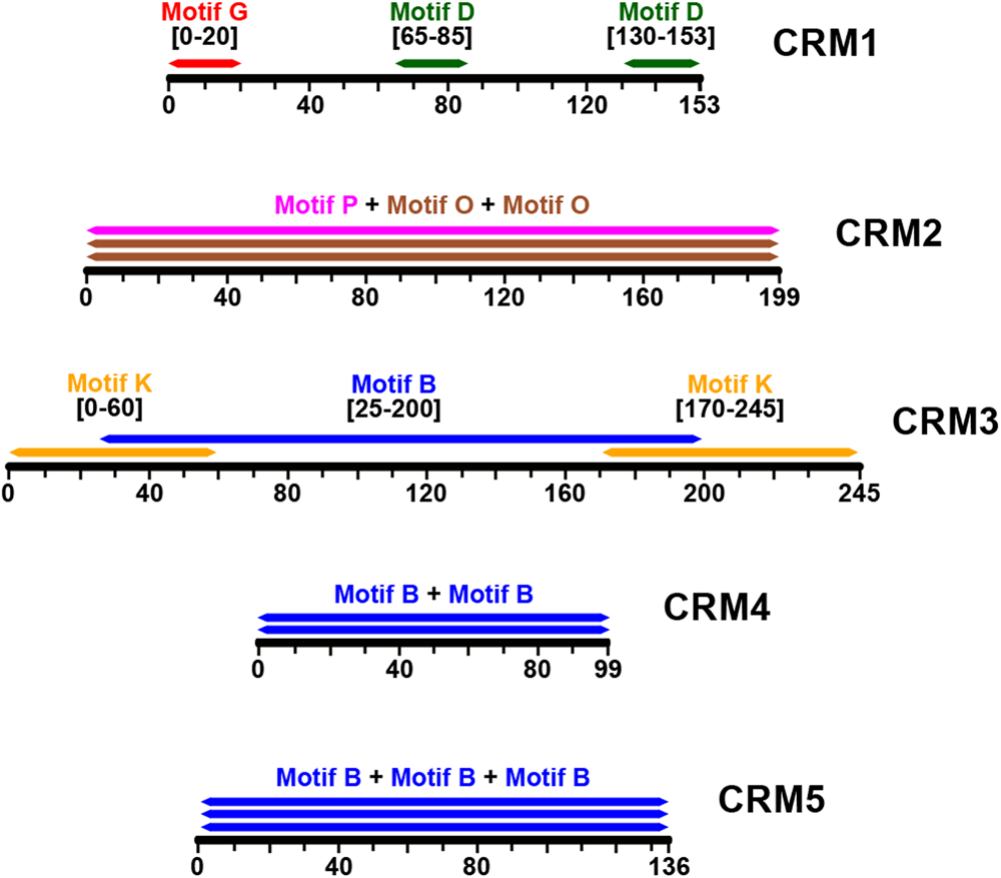
CRMs and their constituent motifs. Each CRM found using a modification of the ReLA algorithm is shown with respective constituent motifs. Each range (X-axis) of the CRMs illustrates the respective sizes. The sub-range for each constituent motif (color-coded bars) is given. CRM2, CRM4, and CRM5 contain their respective constituent motifs in any position across the CRM window.

Commonalities in proximity and organization of the discovered motifs were identified across BS-DR cluster gene promoters. Promoter sequences of BS-DR genes from both IR64 and Nipponbare genomes were converted to a list of position-specific locations of each motif (“motif profile”), which were then aligned using the Regulatory region Local Alignment tool (ReLA)^44^. Sequence segments containing a specific group of motifs, called *cis-*Regulatory Modules (CRMs), were conserved across many BS-DR promoters and were classified in five different groupings, CRM1-5 (Fig. 3, Table 1). Each CRM has a specific window size and occurs throughout the 2 Kb promoter. The relative positioning of the CRM constituent motifs is dispersed (CRM2, CRM4, CRM5) or specific (CRM1, CRM3) (Fig. 3). Constituent motif sub-region specificity in CRM1 and CRM3 was consistent across FA-DR gene promoters and the promoterome (Fig. S4). The five different CRMs show statistical enrichment in FA-DR genes and/or the BS-DR cluster in IR64 and Nipponbare, suggesting their association with the DR (Table 1). The five CRMs described were also tested for enrichment in the other 64 co-expression gene clusters, and some exhibited enrichment in one or more clusters (Table S10). These clusters enriched in CRMs will be useful in future work to test the predicative role of CRMs in finding new BS-DR genes. These clusters, however, were not enriched for both DR characteristics (FA-DR genes and DR-GO terms) as in the BS-DR cluster. Thus, for this work, enrichment in the BS-DR cluster, specifically, will be discussed.

**Table 1:** CRM composition of rice gene promoters.

Genes with CRMs in their promoters				Enrichment tests in DR gene sets^b^	
CRM	Variety	All genes	Orthologous^a^	BS-DR cluster	FA-DR genes
CRM1	IR64	796	678	11 [0.0777]	7 [0.0171]
	Nipponbare	750	670	13 [0.0595]	9 [0.0106]
CRM2	IR64	1,164	947	19 [0.0053]	8 [0.0402]
	Nipponbare	1,020	937	21 [0.0033]	12 [0.0063]
CRM3	IR64	866	714	12 [0.0665]	4 [0.3222]
	Nipponbare	846	751	16 [0.0200]	5 [0.3846]
CRM4	IR64	18,112	13,250	195 [6.577e-06]	66 [0.1725]
	Nipponbare	14,787	12,802	195 [5.658e-05]	76 [0.2424]
CRM5	IR64	5,519	4,011	74 [3.704e-05]	16 [0.7504]
	Nipponbare	4,596	3,948	76 [5.620e-05]	20 [0.7182]

^a^Genes with a given CRM that that exist in both Nipponbare and IR64 varieties.

^b^*P*-values are given in brackets from a test for enrichment in each given set relative to the rest of the promoterome.

CRM1 shows enrichment in FA-DR genes, but not in the BS-DR cluster, whereas CRM2 is enriched in both varieties in both sets of genes. CRM3, which is more prevalent in Nipponbare among strictly the orthogonal set of genes, shows enrichment in Nipponbare in the BS-DR cluster only. CRM4 and CRM5 are both highly enriched in BS-DR genes, but not in FA-DR genes.

Overall, each CRM is associated with BS-DR genes, FA-DR genes, or both gene sets, and thus, their presence in a promoter infers DR involvement. The functional significance of each CRM is illustrated by the unique associations among their respective constituent motifs.

#### -CRM4 and CRM5 – W-box recurrence

Motif B, the pattern found within both CRM4 and CRM5, is in perfect alignment with the core W-box. Both CRMs are highly enriched in the BS-DR cluster of co-expressed genes, and are found in many FA-DR genes (Table 1). The W-box, or WRKY binding site, organizes into groupings of either two (CRM4) or three (CRM5) within a small window (Fig. 3). This suggests that W-boxes tend to work in tandem or repeated patterns. Indeed, some WRKY proteins form homo- and hetero-complexes when binding to promoters, and this happens where there are multiple, closely positioned W-boxes^45^. For instance, *OsWRKY4* is regulated by the binding of OsWRKY80 on the promoter to facilitate resistance to *R*. *solani*, and the W-box sites in the promoter of *OsWRKY4* are in a close-proximity, duplicated pattern^37^. In promoters of Arabidopsis genes involved in Systemic Acquired Resistance (SAR), W-boxes are in tandem, often as duplicates, and are enriched in SAR genes relative to the rest of the genome^46^. In our analysis, W-box (Motif B) occurrence in singlets and duplicates are overrepresented in the BS-DR gene set (Fig. 2, Table 1), thus verifying this trend in rice. While the W-box *cis*-element is in most promoters, its presence in duplicate or triplicate may be a more functional genotype pertaining to BS-DR genes.

#### CRM2 – Two different motifs with interconnected functional roles

Motifs O and P within CRM2 align with functional *cis*-elements found in ethylene- and light-responsive genes, respectively (Figs 2 and 3). Ethylene is a plant hormone that contributes to defense against various pathogens^41^. Photosynthetic genes also play a role in the rice DR and are modulated during infection by pathogens^47–49^. Interestingly, cross-talk exists between ethylene- and light-responsive genes, such as during the process of leaf greening and hypocotyl elongation^50,51^. Perhaps the grouping of *cis-*elements in CRM2 in BS-DR genes is a marker for this intercommunication, thus BS-DR genes with CRM2 can be responsive to both pathways. The identification of CRM2 is a step towards understanding how BS-DR genes with vastly different function intercommunicate, and helps to identify their roles.

#### CRM1 and CRM3 – Putatively involved in production of small-interfering RNAs

**Figure 4:**
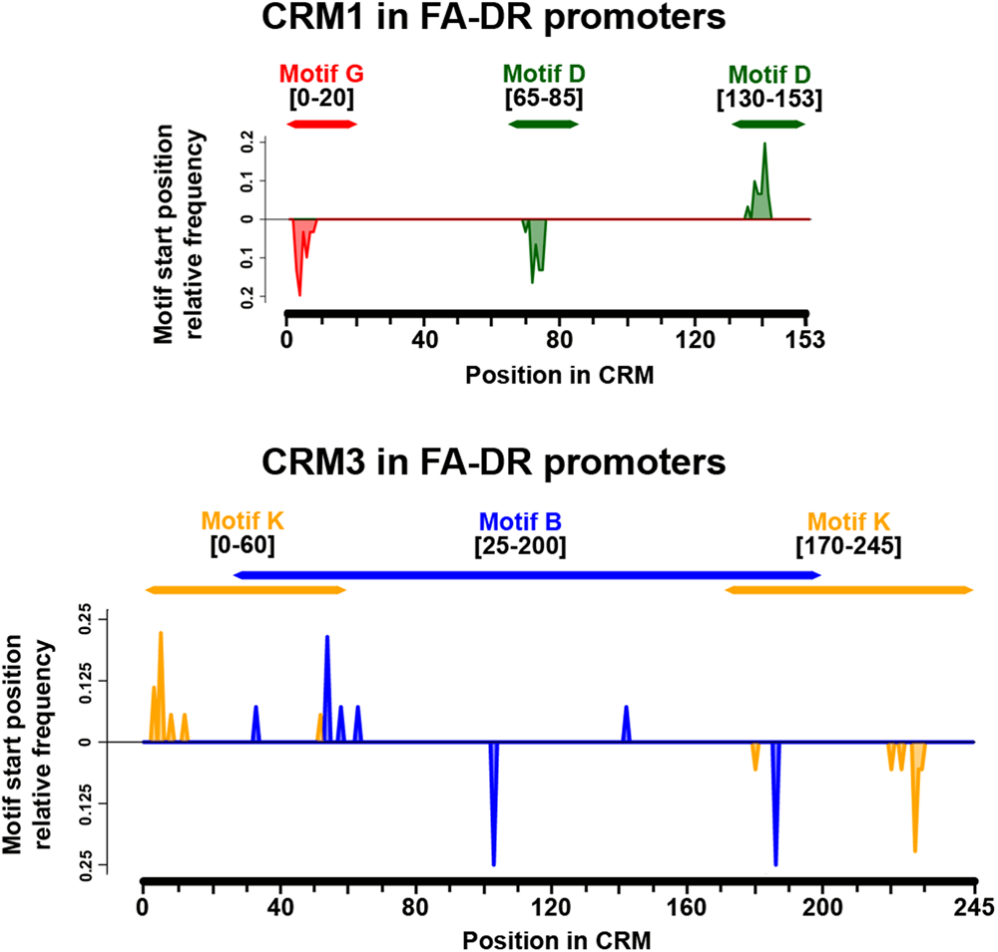
Position specificity of CRM1 and CRM3 constituent motifs. Relative frequency of occurrence (Y-axis) is given for the constituent motifs for both CRMs. Frequency for each motif position across the CRM window was calculated as a ratio of total occurrences of the constituent motif in the CRM found in all FA-DR genes. The strand which the motif is found is given as either below (−strand) or above (+strand) the Y-axis origin.

The constituent motifs within both CRM1 and CRM3 exhibit position specificity across FA-DR promoters and the entire promoterome (Figs 4 and S4). This pattern suggests conservation of all nucleotides within the given windows of CRM1 and CRM3. Consensus sequences were generated for CRM1 and CRM3 by aligning every occurrence of the respective CRM across the Nipponbare promoterome (Fig. S5). The majority of nucleotides within all occurrences of CRM1 and CRM3 show conservation. To identify what type of repetitive sequences CRM1 and CRM3 represent, the consensus sequences were aligned to both known miRNA stem loops (www.mirbase.org) and the Rice Transposable Element (RiTE) database (www.genome.arizona.edu/cgi-bin/rite/index.cgi) (Fig. S6). Both CRMs showed significant (E-value > 1e-10) alignment to segments of known miRNAs and Miniature Inverted-repeat Transposable Elements (MITEs). CRM1 aligned with the sorghum miRNA classified *miR6225*, whereas CRM3 aligned with many members from the rice *miR818* and *miR812* families. Additionally, CRM1 and CRM3 aligned with MITE superfamilies *PIF-Harbinger* and *Tc-Mariner*, respectively, which are both class II Terminal Inverted Repeat (TIR) transposons^52,53^. The secondary structure of putatively transcribed RNA from the CRM1 and CRM3 illustrates self-complementarity similar to the miRNA stem loops (Fig. S7)^54^. The highest scoring alignments were with the MITE sequences, which also form stem loops, and therefore we hypothesize that CRM1 and CRM3 are artifacts of genome-wide transposition events.

**Figure 5:**
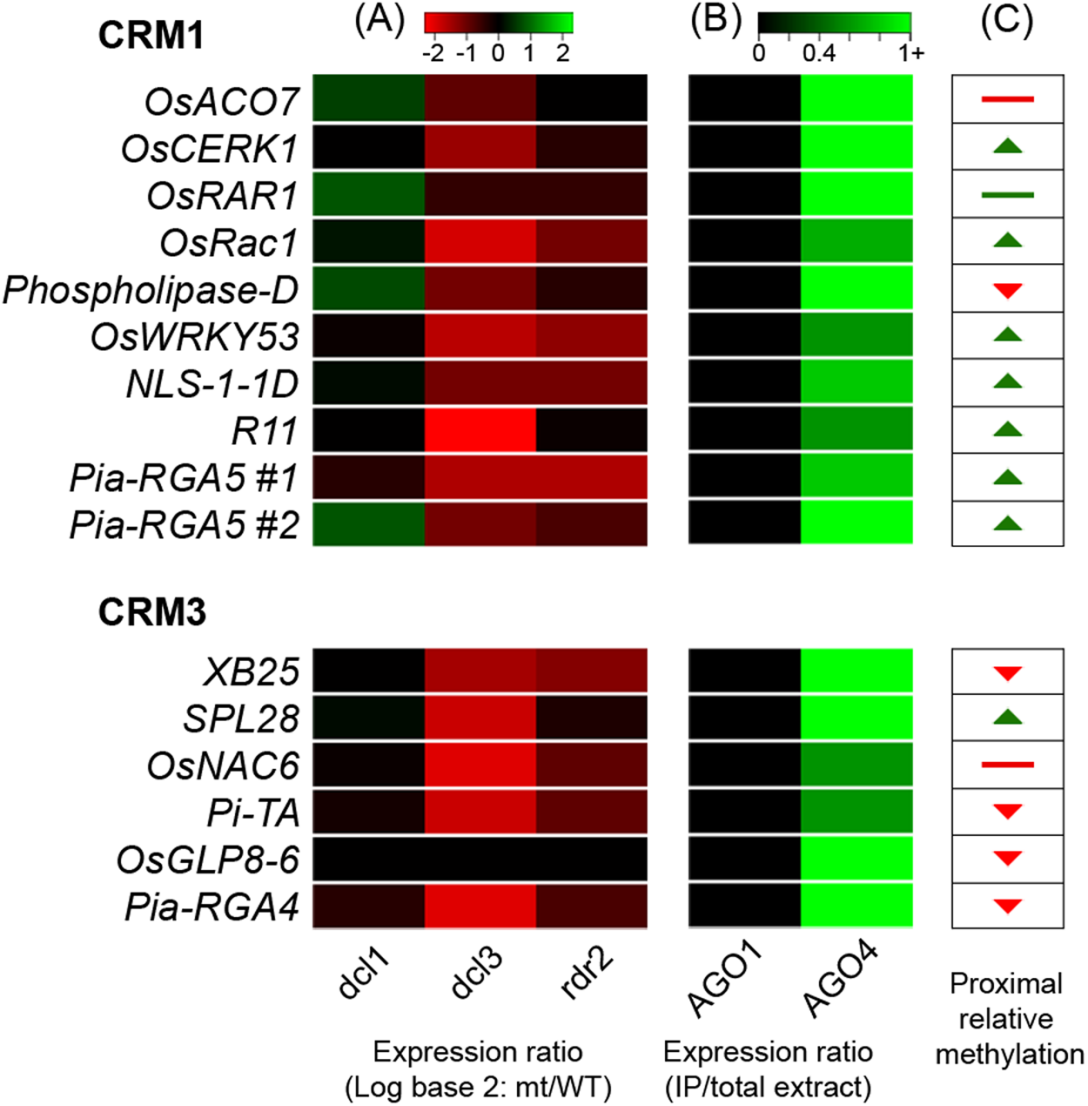
sRNA expression and DNA methylation at FA-DR gene CRM1 and CRM3 loci. Relative sRNA expression (log2 fold change relative to WT) in sRNA processing mutants, *dcl1*, *dcl3*, and *rdr2* (**A**), sRNA abundance ratio of total sRNA extract from AGO1 or AGO4 protein immunopurification experiments (**B**), and relative DNA methylation characteristics (**C**) are shown. Methylation characteristics are based on DNA methylation level in a 1 Kb region surrounding the CRM1 or CRM3 loci. Relative peaks (green upward arrow), valleys or decreases (red downward arrow), complete absence (red line), or consistent methylation (green line) are given for each locus.

Since CRM1 and CRM3 sequences show characteristics of a repeat element, it is possible that expression of small RNAs (sRNAs) at these loci affects gene regulation. Rice sRNA expression data at each CRM1 or CRM3 locus in FA-DR genes was extracted from a sRNA Illumina SBS sequencing database^55^. Across multiple experimental libraries, relative peaks of sRNA expression occur within each locus of CRM1 or CRM3 (Fig. S8). To discover any possible epigenetic function of sRNA produced at the CRM loci, sRNA counts were examined from individual experiments in the sRNA database (Fig. 5, Table S11). The majority of sRNA produced at the FA-DR promoter CRM loci were 24 nucleotides in length. Some CRM loci produced both 21 and 24 nt sRNAs in abundance, such as loci for *Phospholipase-D*, *R11*, and *NLS-1-1D* (Table S11). In the first data set examined, total sRNA reads were obtained in processing mutants, Dicer-Like 3 (*dcl3*), Dicer-Like 1 (*dcl1*), and RNA-dependent RNA polymerase II (*rdr2*) and were compared relative to the wild-type (Fig. 5A, Table S11). In *dcl3* and *rdr2* mutants, 24 nt sRNAs were reduced compared to wild-type, but increased or were unaffected in *dcl1* mutants. Conversely, the 21 nt sRNAs from a few loci generally decreased in *dcl1*, but increased in *rdr2* and *dcl3* mutants relative to wild-type (Table S11). In the next data set, total sRNA was obtained from wild-type plant tissue or the sRNA accompanying the immunopurification of Argonaute-1 (AGO1) or AGO4 protein classes (Fig. 5B, Table S11). In most loci for both CRM1 and CRM3 in DR genes, sRNAs were 24 nt in size and immunoprecipitated with the class of AGO4 but not AGO1 proteins (Data extracted from^56,57^) (Fig. 5B). The production of 24 nt sRNA is dominant across CRM1 and CRM3 loci, and these sRNAs show an association with AGO1, DCL3 and RDR2 proteins, which are involved in the processing of and interaction with small-interfering RNAs (siRNAs)^57^. Thus, CRM1 and CRM3 may function in producing these types of regulatory siRNAs.

#### CRM1 and CRM3 – Indicators of epigenetic modification

To shed light on possible epigenetic activity, methylation at each of the CRM1 and CRM3 loci was examined from publicly available rice bisulfite sequencing data (Fig. S8) (mpss.danforthcenter.org)^55^. Within a 1 Kb region surrounding each CRM locus, a relative methylation increase or decrease was observed at these CRMs. For CRM1 loci, six out of nine FA-DR genes that contain CRM1 in their promoters, *OsCERK1*, *OsWRKY53*, *OsRac1*, *R11*, *NLS1-1D*, and both instances in *Pia-RGA5*, show relative increases in methylation compared to the surrounding DNA (Figs 5C and S8). One gene, *OsRAR1*, exhibits consistent methylation across the region. The remaining two FA-DR loci with CRM1, *OsAGO7* and *Phospholipase-D*, respectively, show no methylation, or a valley where methylation markedly decreases relative to the surrounding DNA. In CRM3 from FA-DR gene promoters, four of the six total loci, *XB25*, *OsGLP8-6*, *Pia-RGA4*, and *Pi-TA*, show a methylation valley. The gene *SPL28* has a small peak in methylation at CRM3, and *OsNAC6* shows no methylation in the region. These combined results indicate that methylation/de-methylation that is occurring is specific to the CRM1 and CRM3 sites, and that these sites play a role in dictating methylation activity due to an observed difference between the regions within and outside each CRM. CRM1 sites in FA-DR gene promoters tend to show peaks in methylation, whereas CRM3 sites show relative valleys, suggesting functional variation in methylation activity. This methylation data is from Nipponbare plants under no stress; thus, further experimentation is needed to determine active methylation/de-methylation of CRM1 and CRM3 loci during pathogen infection.

We hypothesize that the sRNAs transcribed in each CRM1 and CRM3 locus are predominantly producing siRNAs and participating in *cis*-acting RNA-directed DNA methylation (RdDM) for a variety of reasons. First, the majority of reads are 24 nt in length and immunoprecipitate with AGO4 proteins, which is indicative of the siRNA processing pathway (Fig. 5, Table S11)^56–58^. Secondly, DCL1 is the protein involved in 21 nt miRNA processing, and when mutated, *dcl1* lines do not show changes in accumulation of 24 nt sRNAs produced at CRM loci (Fig. 5A)^57,59^. Conversely, mutation of RDR2 or DCL3, enzymes responsible for processing and amplification of siRNAs, results in decreased amounts of 24 nt reads produced relative to the wild-type, meaning these proteins are critical in the production of these CRM-derived sRNAs (Fig. 5A). Finally, methylation/de-methylation is altered at the CRMs relative to surrounding DNA, illustrating that the siRNAs produced at CRM1 and CRM3 sites could be participating in RdDM in a *cis*-regulatory manner.

The involvement of RdDM in disease resistance is well-studied in plants. A demethylating agent, 5-azadeoxycytidine, enhances resistance of rice to *X*. *oryzae* pv. *oryzae*^60^. In *Arabidopsis*, active DNA de-methylation occurs during the resistant response to *Pseudomonas syringae*, and many DR genes with repeat elements in their promoters are affected by epigenetic processes^61^. One gene, *WRKY22*, is methylated in the promoter in the basal state, but actively demethylates during pathogen infection, allowing for transcription factors to bind and activate the gene^61^. Conversely, active DNA methylation is important for defense against the tumor-inducing *Agrobacterium tumefaciens* and necrotrophic fungi^62,63^.

Many of the FA-DR genes that contain CRM1 or CRM3 within their respective promoters exhibit polymorphisms between haplotypes of IR64 and Nipponbare (Fig. S9). This evidence suggests functional differences that result from promoter variation of CRM1/CRM3 through deletion or positional shifting. These haplotype variants are key to understanding this mechanism. Further research is needed to determine if these promoter elements are key RdDM mechanisms for broad-scale genomic regulation and disease resistance.

### CRMs occur in BS-DR genes located in disease resistance Quantitative Trait Loci (QTL)

**Figure 6:**
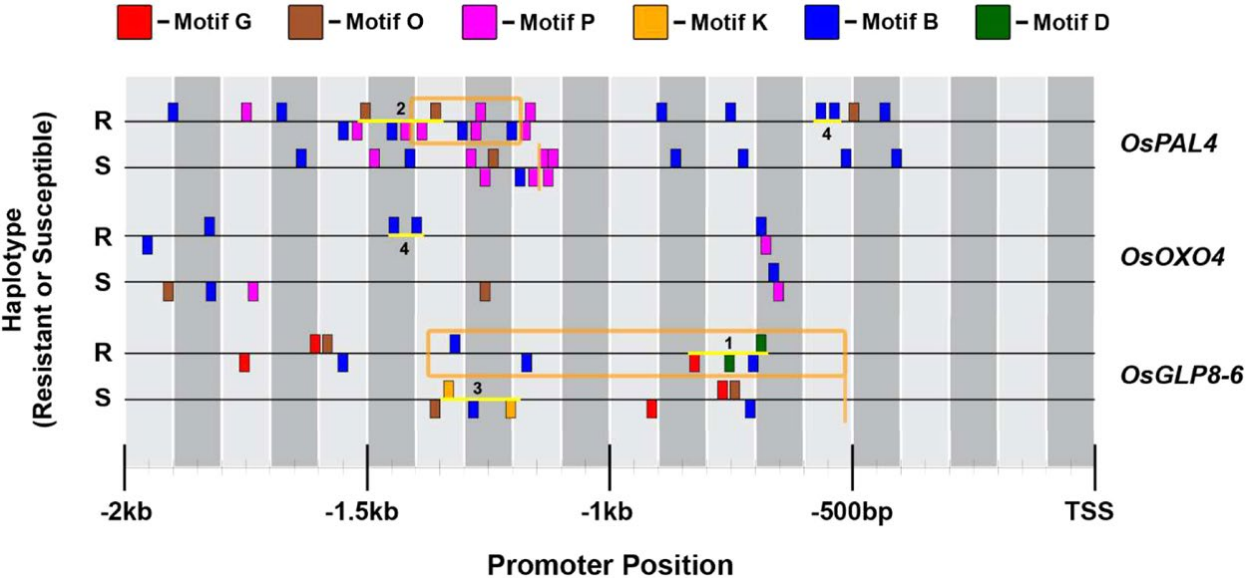
CRM motif profiles of FA-DR genes in resistant and susceptible QTL haplotypes. CRM promoter motifs and labelled CRMs (yellow lines) within resistant (R) or susceptible (S) QTL haplotypes for *OsPAL4* (R: IR64, S: Azucena), *OsOXO4* (R: Moroberekan, S: Vandana), and *OsGLP8-6* (R: SHZ-2, S: LTH). Motifs on plus or minus strand are above or below horizontal lines, respectively. The resistant donor of *OsOXO4*, Moroberekan, does not have available genome sequence, thus another japonica sub-population variety, Nipponbare, was used. Indels are shown as highlighted orange boxes (insertion) or vertical lines (deletion site).

BS-DR genes located within disease resistance QTL contribute to resistance^6,12,13,26,27^. Three different DR gene families within disease resistance QTL contain CRMs; the Phenylalanine Ammonia Lyases (*OsPAL*s), the Oxalate Oxidases (*OsOXO*s), and the Germin-Like Proteins (*OsGLP*s) (Fig. S10). Some members of these gene families confer resistance to diverse pathogens, and these genes’ promoters contain CRM polymorphisms in the resistance haplotypes (Table S12). Intriguingly, the family members not contributing to the phenotype lack CRMs (Figs 6 and S10).

Four *OsPAL* gene family members co-localize with a resistance QTL on chromosome 2, and of those four members, *OsPAL4* is the gene critical for the resistance phenotype^13^. The CRM profile of the *OsPAL4* promoter differs between resistant and susceptible QTL donor haplotypes (Fig. 6). In susceptible haplotype (Azucena), there is a 229 bp deletion at position −1,141 from the TSS, removing two constituent motifs from CRM2; CRM2 remains intact in the resistant haplotype (IR64). Additionally, CRM4 in the IR64 promoter located at position −532 has a single nucleotide deletion at the first Motif B in the susceptible haplotype, therefore disrupting the CRM. The other members of the *OsPAL* gene family on chromosome 2, *OsPAL1*, *OsPAL2*, and *OsPAL3*, are not involved in resistance and show no difference in promoter motif structure (Fig. S10A)^13^. The coding sequence of *OsPAL4* does not differ between the resistant and susceptible haplotypes^13^. Thus, the promoter polymorphisms that alter CRM2 and CRM4 are likely causal elements of resistance.

The family of *OsOXO* genes in a disease resistance QTL contain one BS-DR gene (*OsOXO4*) and three other family members with no DR functionality, *OsOXO1*, *OsOXO2*, and *OsOXO3*. The genes *OsOXO1-3* are not expressed during *M*. *oryzae* infection, whereas *OsOXO4* is expressed, and it is more highly expressed in the resistant haplotype during infection^27^. *OsOXO4* contains CRM4 in the resistant haplotype promoter compared to the susceptible allele (Fig. 6). No significant differences in promoter structure and CRM composition were observed in *OsOXO1* or *OsOXO2* promoters (Fig. S10B). In contrast, CRM5 occurs in the susceptible haplotype in the non-DR *OsOXO3*, suggesting that enhanced expression of this locus due to CRM5 may increase susceptibility or play a role in different stresses or developmental stages. The differences in promoter, not coding gene sequence, were previously confirmed to be a causal element of resistance achieved with *OsOXO4*^27^, and the unique occurrence of CRM4 in the resistant variety supports this hypothesis.

The *OsGLP8* family resides in a disease resistance QTL consisting of 12 genes, with *OsGLP8-6* as a key contributor^12,26^. *OsGLP8-6* has a large insertion (856 bp) in the resistant haplotype promoter at position −517 that is associated with higher and earlier expression in response to pathogen inoculation^26^ (Fig. 6). This insertion contains CRM1 at the 3′ end, as well as three instances of Motif B, which are W-box motifs (Fig. 6). The CRM3 occurrence in the susceptible haplotype is unchanged in the resistant haplotype, but it is upstream of the visual window of −2 Kb due to the promoter insertion. Since CRM1 and CRM3, putative epigenetic elements, occur in the promoter of *OsGLP8-6*, with CRM1 only found in the resistant haplotype (within the insertion), the displacement of CRM3 and introduction of CRM1 in the resistant haplotype could be the reason for faster and higher expression of *OsGLP8-*6^26^. However, sRNA production and local methylation are not pronounced at the CRM3 site in the *OsGLP8-6* promoter (Fig. 5). Other FA-DR genes with CRM3 in their respective promoters contain a larger, palindromic motif profile with complementary flanking sites of Motif B as compared to the truncated version of CRM3 seen with *OsGLP8-6* (Fig. S10B). This may explain the lack of sRNA expression at the CRM3 locus in the *OsGLP8-6* promoter (Fig. 5). Additionally, the *OsGLP8-6* CRM3 secondary structure exhibits more intermittent self-complementarity as compared to other CRM stem loop structures (Fig. S10). Other *OsGLP8* family members within the resistance QTL have been functionally validated to contribute to resistance by gene silencing (*OsGLP8-6*, *OsGLP8-7*, *OsGLP8-9*)^12^. All of these genes contain an instance of at least one CRM in their promoters, and show polymorphisms between resistant and susceptible haplotypes (Fig. S10C). Family members that do not contribute to the DR in gene silencing experiments, *OsGLP8-2*, *OsGLP8-3*, and *OsGLP8-12*, lack CRM polymorphisms (Fig. S10C).

### Translating CRMs into a genome-wide prediction of BS-DR genes

The CRMs described herein were discovered in a *de novo* fashion, starting with a set of co-expressed BS-DR gene promoters combined across two genomes, IR64 and Nipponbare, discovering small motifs enriched within these promoters, and finally uncovering a spatial patterning of these motifs across BS-DR promoters. Some CRMs (CRM2, CRM4 and CRM5) were indicative of transcription factor binding in cooperation, while others revealed a longer sequence conservation across promoters (CRM1 and CRM3) that infer epigenetic activity influencing BS-DR gene regulation.

We provide three examples of CRMs within promoters of resistance QTL-associated BS-DR genes, and not within “Non-DR” gene promoters, to support the predictive potential of CRMs to illustrate the effectiveness of BS-DR gene regulation. Our analysis shows that differences in the promoters of BS-DR genes between resistant and susceptible haplotypes include the mutation and/or structural change of CRMs (Figs 6 and S10). The ability for DR genes to modulate transcription during infection may be influenced by the regulatory mechanisms dictated by these CRM genotypes.

The understanding both that common CRMs are present and that their differences between rice varieties can discern the resistance phenotype can allow us to predict novel BS-DR genes across the genome. The criteria for selection of parents and the next generations in breeding programs could take advantage of this knowledge. Thus, knowledge of promoter composition, and how important that is for a strong basal resistance, will allow selection of varieties enriched in the broad-spectrum DR across the genome.

## Materials and Methods

### Broad-spectrum defense response transcriptome data

A collection of publicly available transcriptome data from NCBI GEO (www.ncbi.nlm.nih.gov/geo/) and recent publications were mined to identify rice defense response (DR) genes that are co-regulated across various diseases and treatments (Table S1), filtering for “treatment versus mock” scenarios of resistant interactions. Affymetrix probe results files (CELs) were processed for each treatment-control study with the R package, Affy, which utilizes Robust Multi-Array (RMA) background correction and quantile normalization^64^. Affymetrix probes were matched to the MSU7 Nipponbare rice reference genome^65^ using the sequence alignment software, Vmatch (www.vmatch.de). Agilent microarray data was processed using the NCBI GEO software, GEO2R (www.ncbi.nlm.nih.gov/geo/geo2r/). Illumina RNA sequencing results were checked for quality using FastQC (Babraham Bioinformatics). Sequence reads were preprocessed by removing the first 15 base adapter sequences, then trimming the ends with parameters of a minimum score of 20 and minimum read length of 20 using the fastx toolkit (hannonlab.cshl.edu/fastx_toolkit/index.html). The pre-processed fastQ files were aligned to the representative protein coding mRNAs from the MSU7 Nipponbare reference genome^65^, and the resulting “.bam” file was assembled into transcripts and annotated. These alignment processes, along with calculating differential expression ratios were accomplished using TopHat, Cufflinks, Cuffdiff, and Cuffmerge, sequentially, from the Tuxedo analysis package^66^.

To analyze the differential expression data sets across various platforms, each set was converted to a log base 2 scale fold change then normalized to make cross-comparison feasible. The 44 individual expression data sets were centered based on their respective medians within the 3*(interquartile range). Data was clipped by removing expression values that were outside of 3*(interquartile range). Normalization was performed using a min-max linear transformation within the range of −1 to 1 (Fig. S1).

### DR gene co-expression analysis

Genes with missing data for greater than 10% of the expression studies were excluded. Otherwise, missing values were imputed using the median expression of the associated experiment. Pearson correlation, followed by average linkage hierarchical clustering of the distance measure, 1-correlation, was performed using the R packages “cor” and “hclust”, respectively (www.R-project.org). Branch cutting of the resultant dendrogram was done using various parameter settings and algorithms of the dynamic tree cut method, an improvement from using a fixed cut height^67^. Specifically, the “cutreeHybrid” R-script was used with default parameters aside from an altered minimum cluster gap ranging from 0.15 to 0.40, or the “cutreeDynamic” R-script with “deepSplit” parameters, 0, 1, 2, 3, True, or False. Gene Ontology (GO) term and known DR gene enrichment analysis of co-expressed gene clusters utilized the Fisher exact test, with Benjamini-Hochberg correction and a False Discovery Rate (FDR) of 0.05^68^. Choosing the dendrogram branch cutting parameters to produce clusters for subsequent analysis involved finding the method which maximized Dunn Index and Connectivity, minimized Silhouette Width, and contained the most DR-GO and DR gene enriched clusters (Fig. S2, Table S3). GO terms were taken from the plant GOSlim database aligned to the rice genome (rice.plantbiology.msu.edu/index.shtml) (Table S4). A list of known defense genes, functionally associated (FA) with plant defense (FA-DR genes), in the rice genome was compiled using recent literature and publicly available databases, the Overview of functionally characterized Genes in Rice Online (OGRO) database and the Kansas State University Rice Defense Gene Collection (www.k-state.edu/ksudgc/) (Table S5)^69^.

### Promoterome construction

The promoterome of the rice variety Nipponbare was constructed using the MSU7 rice reference annotation^65^ and a custom python-script. Only the representative gene model (contained furthest upstream 5′ UTR) was chosen for each locus, and transposable element-encoding genes were omitted. Transcription Start Sites (TSSs) were assigned to the −1 position from the start of the genes. Promoters were limited to 2 Kb upstream of the assigned TSS, unless that segment overlapped with a neighboring gene sequence, in which case the 5′ end of the promoter was truncated to the end of that respective gene. After this step, promoters shorter than 50 bp were removed from the analysis due to constraints of the CRM-finding pipeline. Promoters for rice variety IR64 were constructed using the genome sequence and annotation recently developed (http://schatzlab.cshl.edu/data/ir64/). To ensure accurate annotation of IR64 genes, gene orthologs were identified between IR64 and Nipponbare based on 90% similarity in full gene sequence. Accurate TSS positions in the IR64 orthologs were obtained by aligning Nipponbare gene sequences to IR64 contigs using BLAT on linux command line at default parameters aside from max Intron length of 600 (genome.ucsc.edu/cgi-bin/hgBlat?command = start). Any other IR64 annotated genes that did not have an orthologous Nipponbare gene were included in the IR64 gene set if they did not overlap with previously identified orthologs. Promoters from the IR64 contigs were extracted, truncated, and filtered as described above. Consistency in promoter positioning and structure was verified using a graphical representation of the nucleotide tallies at each relative position in each varieties’ promoterome using an R-script.

### Sequence motif finding

Putative *cis*-elements *de novo* were identified using an ensemble approach with multiple motif-finding algorithms to search the promoteromes of the co-expressed broad-spectrum defense response (BS-DR) gene cluster derived from Nipponbare and IR64. The application gimmemotifs was modified to be used as a backbone for running component algorithms simultaneously^34^. Component algorithms were Mdmodule, MEME, Weeder, MotifSampler, trawler, Improbizer, BioProspector, AMD, Homer, and GADEM^70–79^. Sequences of all promoters from each variety less those of the genes in the BS-DR cluster were used as background sequences when required per component algorithm. Default parameters for each algorithm were used aside from certain universal parameters: (1) size range between 6 and 15 bp, (2) search both plus and minus strand, and (3.) output only top 15 motifs. Each motif Position Frequency Matrix (PFM) obtained from the multiple algorithm runs was tested for enrichment in the BS-DR cluster relative to the rest of the genome, with a cutoff threshold of 85% similarity of PFM to query sequence. The enrichment test was done using a “with or without” score for each promoter in IR64 or Nipponbare, and a Fisher exact test with Bonferroni *P*-value correction. Many of the motifs deemed as enriched were highly similar and came from different motif-finding algorithms. Thus, matching motifs were combined into one using a Weighted Information Content (WIC) score and “iterative clustering”^34^. Clustered motifs were tested again for enrichment in the BS-DR cluster due to the changes imposed from motif merging (Fig. 2). Comparison of these *de novo* motifs to known *cis-*elements was done using the program, TOMTOM, of the MEME suite^80^. A list of known *cis*-elements was generated by combining the databases, PLACE (www.dna.affrc.go.jp/PLACE), atcisDB (arabidopsis.med.ohio-state.edu/AtcisDB), and TRANSFAC-plants (gene-regulation.com/pub/databases.html).

### Searching for putative cis-Regulatory Modules (CRMs)

CRMs were found using a window-based, pairwise alignment approach of motifs in promoters of BS-DR cluster genes from IR64 or Nipponbare. This was accomplished with the Regulatory region Local Alignment tool (ReLA), which utilizes a Smith-Waterman algorithm on sequence motif profiles^44^. The program was executed for both Nipponbare and IR64 BS-DR cluster promoters, with two separate size restrictions of the CRM search window: >50 nt, or >200 nt. Due to restrictions of the alignment method, ReLA uses one promoter as a reference to align to each other promoter, then outputs the promoters that match that reference promoter motif profile. Thus, ReLA was run 385 times, using a different BS-DR cluster promoter as the reference each time. From all runs of the program, the reference motif profiles that included the highest number of BS-DR promoters aligned with the test promoter (>10) were selected as putative CRMs. Enrichment of each independent CRM in promoters of BS-DR cluster genes or known DR genes was determined using a Fisher Exact test of the genes with CRMs in the BS-DR cluster versus the rest of the promoterome. Visualizations of CRMs and constituent motifs in promoters of QTL-based DR genes were done using a python-script, and the PyX package (pyx.sourceforge.net). Motif densities in each CRM were calculated from the ratio of total occurrences of each motif start point for each nucleotide position in the CRM window, and plotted using an R-script.

### Classification of CRM1 and CRM3 sequences

For each respective CRM1 and CRM3, all occurrences across the Nipponbare promoterome were extracted using a python-script and aligned using a command line version of Clustal Omega^81^. The alignment was converted into a Hidden Markov Model profile using *HMMer* (HMMer.org). The consensus sequences for CRM1 and CRM3 were then generated using percent occurrence values for each nucleotide at each position along the HMM profile using *HMMemit* (HMMer.org). Consensus sequences were searched using the BLAST utility in miRBase (www.mirbase.org) against all stem loop sequences, and in the RITE database (www.genome.arizona.edu/cgi-bin/rite/index.cgi) for all repeat elements of rice. Putative secondary structure of RNAs produced from CRM consensus sequences was accomplished using the RNAfold webserver (rna.tbi.univie.ac.at/cgi-bin/RNAWebSuite/RNAfold.cgi).

Using a publicly available Illumina SBS small RNA sequencing database, chromosomal coordinates of each CRM1 or CRM3 occurrence in known DR gene promoters were queried for total rice sRNA reads mapped to the regions (mpss.danforthcenter.org/)^55^. Study specific analyses were extracted to look at AGO immunoprecipitation and sRNA processing mutants from the database^56,57^. Methylation results from bisulfite sequencing were extracted as total methylated reads from all experiments using Nipponbare in various tissue types (mpss.danforthcenter.org/~apps/DNA_Met/public/RICE_met/)^55^.

## Supplementary information

Supplementary information

Table S1

Table S2

Table S3

Table S4

Table S5

Table S6

Table S7

Table S8

Table S9

Table S10

Table S11

Table S12

## Data Availability

All data generated or analyzed during this study are included in this published article (and its Supplementary Information files).
