# Supplementary material for: Shared cis-regulatory architecture identified across defense response genes is associated with broad-spectrum quantitative resistance in rice
Source: Sci Rep. 2019 Feb 7;9:1536. doi: 10.1038/s41598-018-38195-x (PMC6367480; doi:10.1038/s41598-018-38195-x)
Supplement: Supplementary file 1 — Supplementary information [file 41598_2018_38195_MOESM1_ESM.pdf]

Supplementary Information

Shared *cis*-regulatory architecture identified across defense response genes is associated with broad-spectrum quantitative resistance in rice

Bradley W. Tonnessen[1], Ana M. Bossa-Castro[1], Ramil Mauleon[2], Nickolai Alexandrov[2], Jan E. Leach[1]

[1]Colorado State University, Fort Collins, CO, USA
[2]International Rice Research Institute, Philippines

This file includes:
Supplementary information, Figures S1-S10
Supplementary information, Table legends S1-S12

Supplementary information, FIGURES

Figure S1.

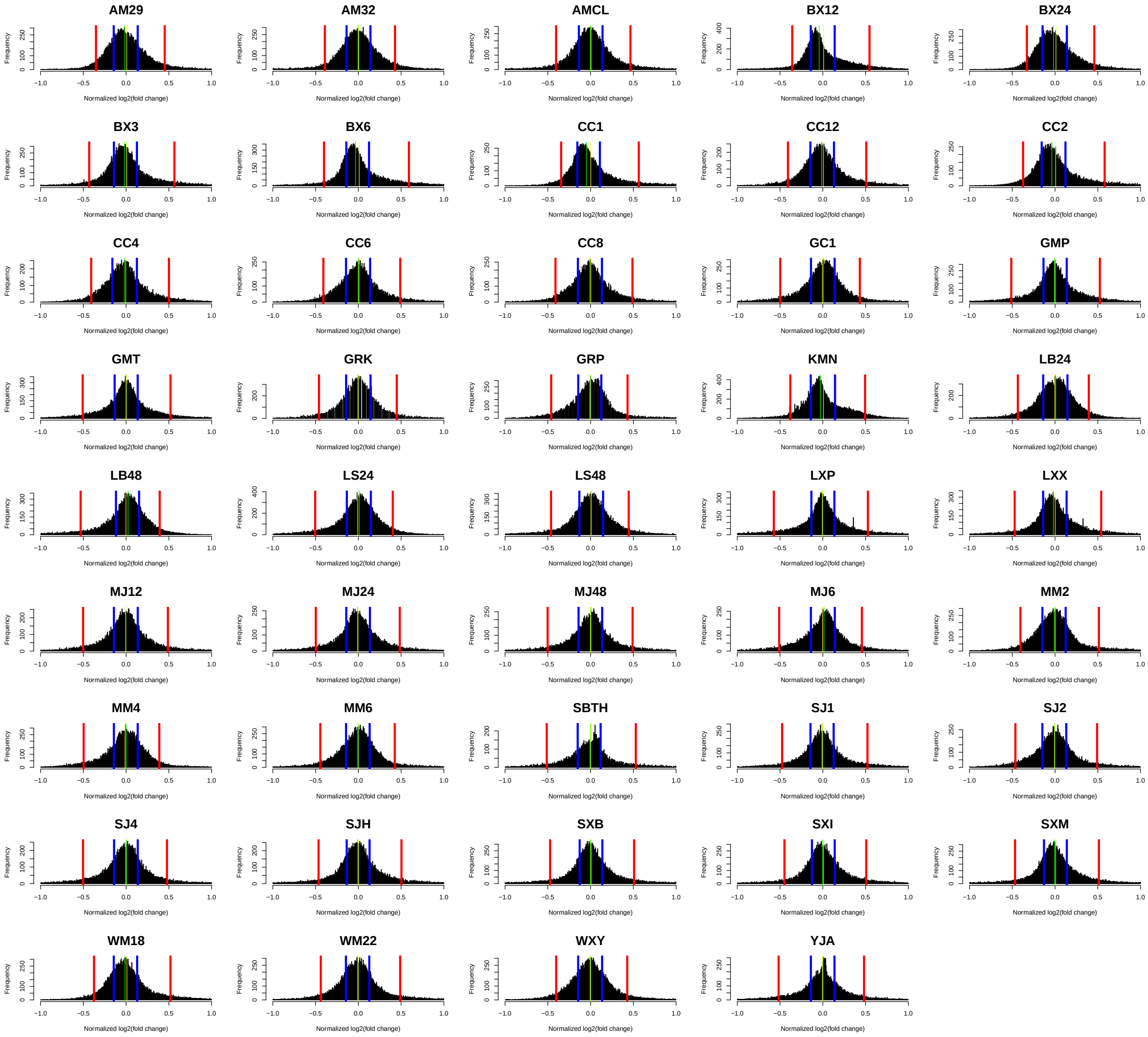

$$X_{new} = \left(\frac{X - \min X}{\max X - \min X} \times 2 \right) - 1$$

Figure S1. Post-processing expression data histograms. The distributions of scaled, centered, and normalized log base 2 fold change expression values for each experiment used in the co-expression analysis. Red lines show the outer 5% quantiles. Blue lines are marking the outer 25% quartiles. The median and mean are given as green and yellow lines, respectively. Normalization using the min-max linear transformation given below. Normalization was done separately for each individual experimental study. The vector X is the original scaled and centered expression data, X_{new} is the vector of expression values used for the analysis going forward.

Figure S2.

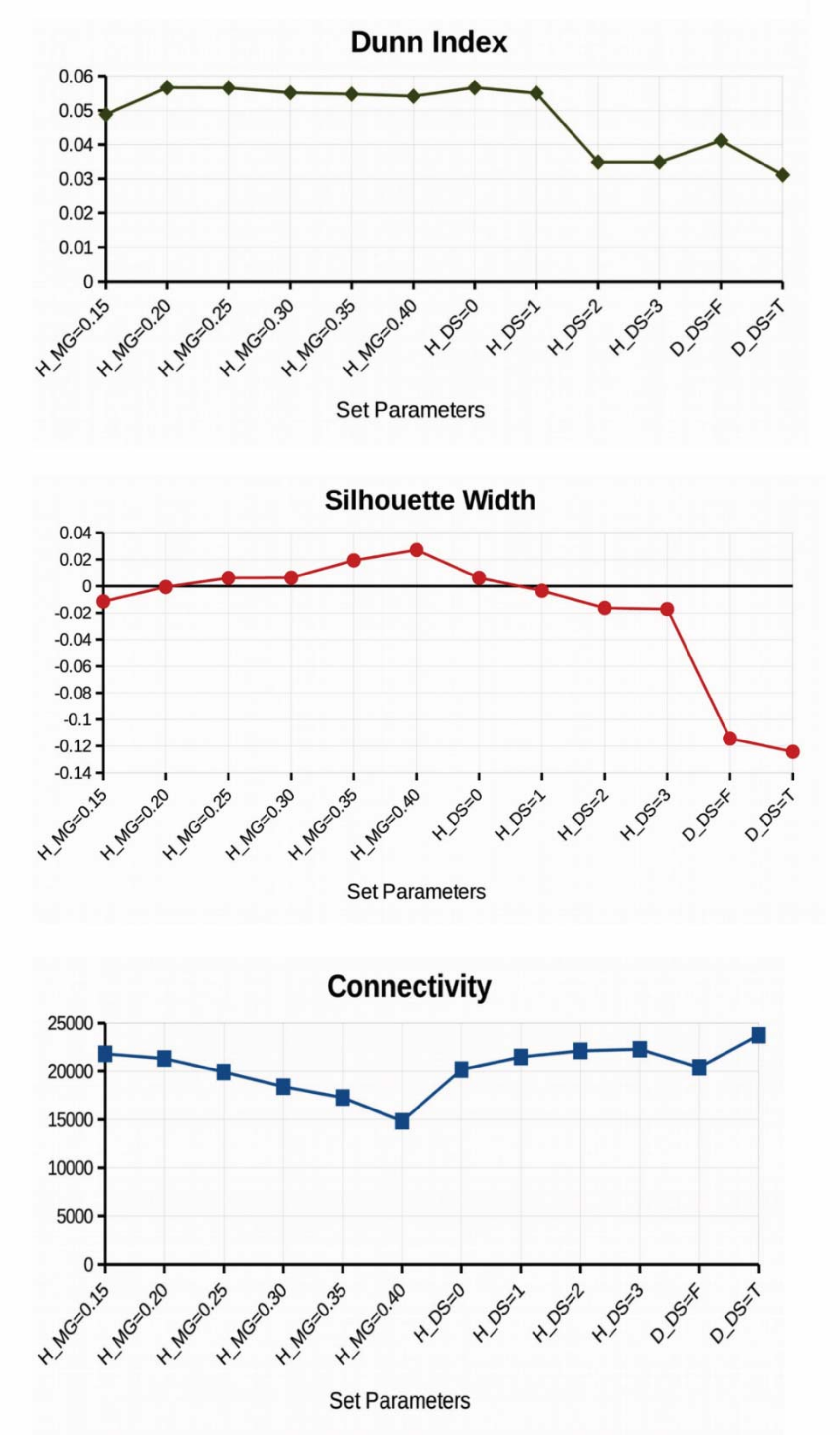

Figure S2. Cluster validation plots. Graphical representation of the different scoring measures for tree cutting methods of the co-expression dendrogram. Different scoring measures were the Dunn Index, Sillhouette Width, and Connectivity between clusters. All calculations were done as described in Xu et al 2009. Chosen parameter set: "H_MG=0.25". [Xu, R. & Wunsch, D. *Clustering, series on computational intelligence*. (Wiley-IEEE Press, 2008).]

Figure S3.

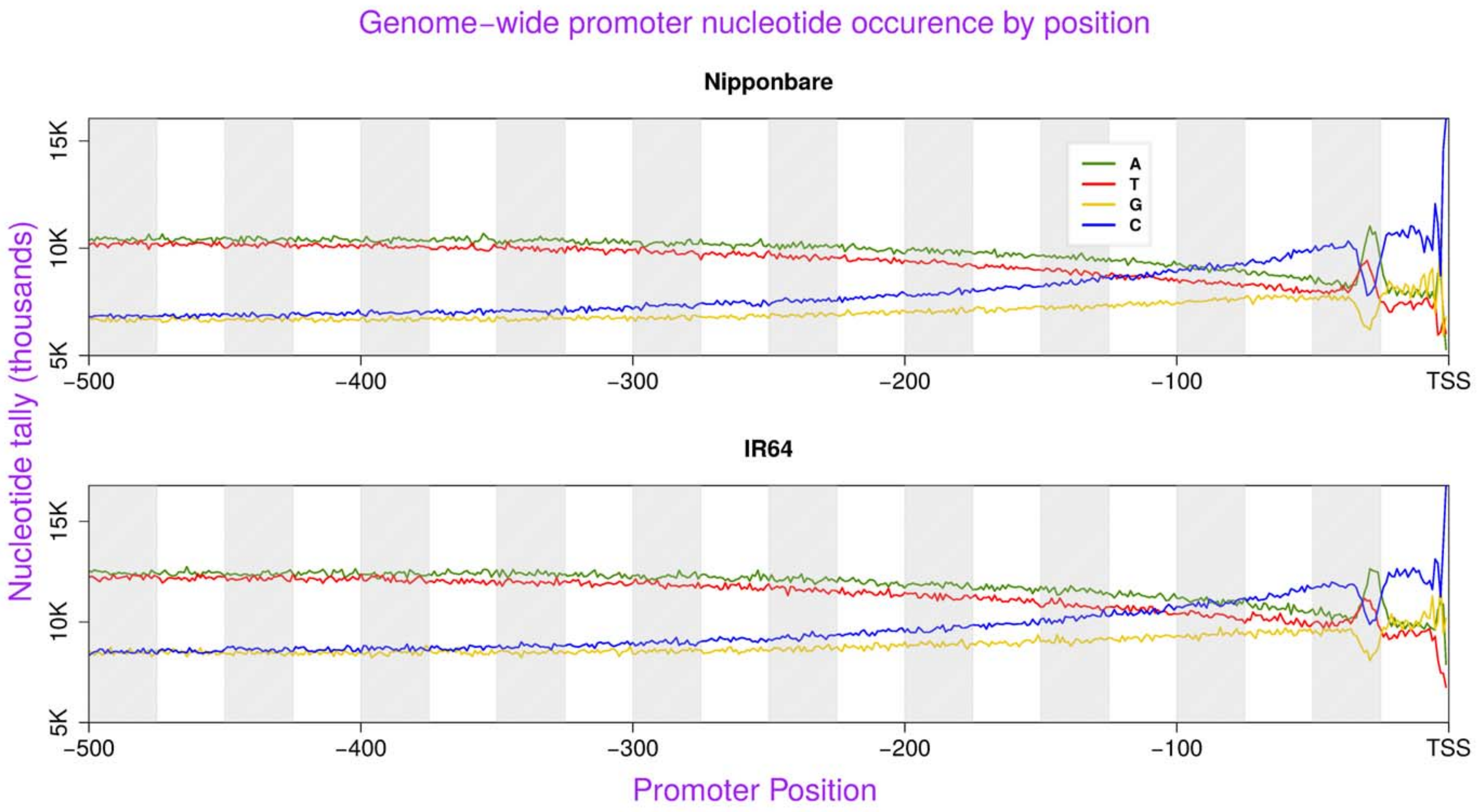

Figure S3. Genome-wide promoter nucleotide occurrence by position. Using the promoteromes from both IR64 and Nipponbare rice varieties, nucleotides were tallied for each position along the promoter from the Transcription Start Site to -500 bases upstream. There are a total of 36,121 promoters in the Nipponbare set, and 43,523 promoters in IR64. For each nucleotide positon along the X-axis, the number of promoters with A (green), T (red), G (yellow), or C (blue) is given on the Y-axis.

Figure S4.

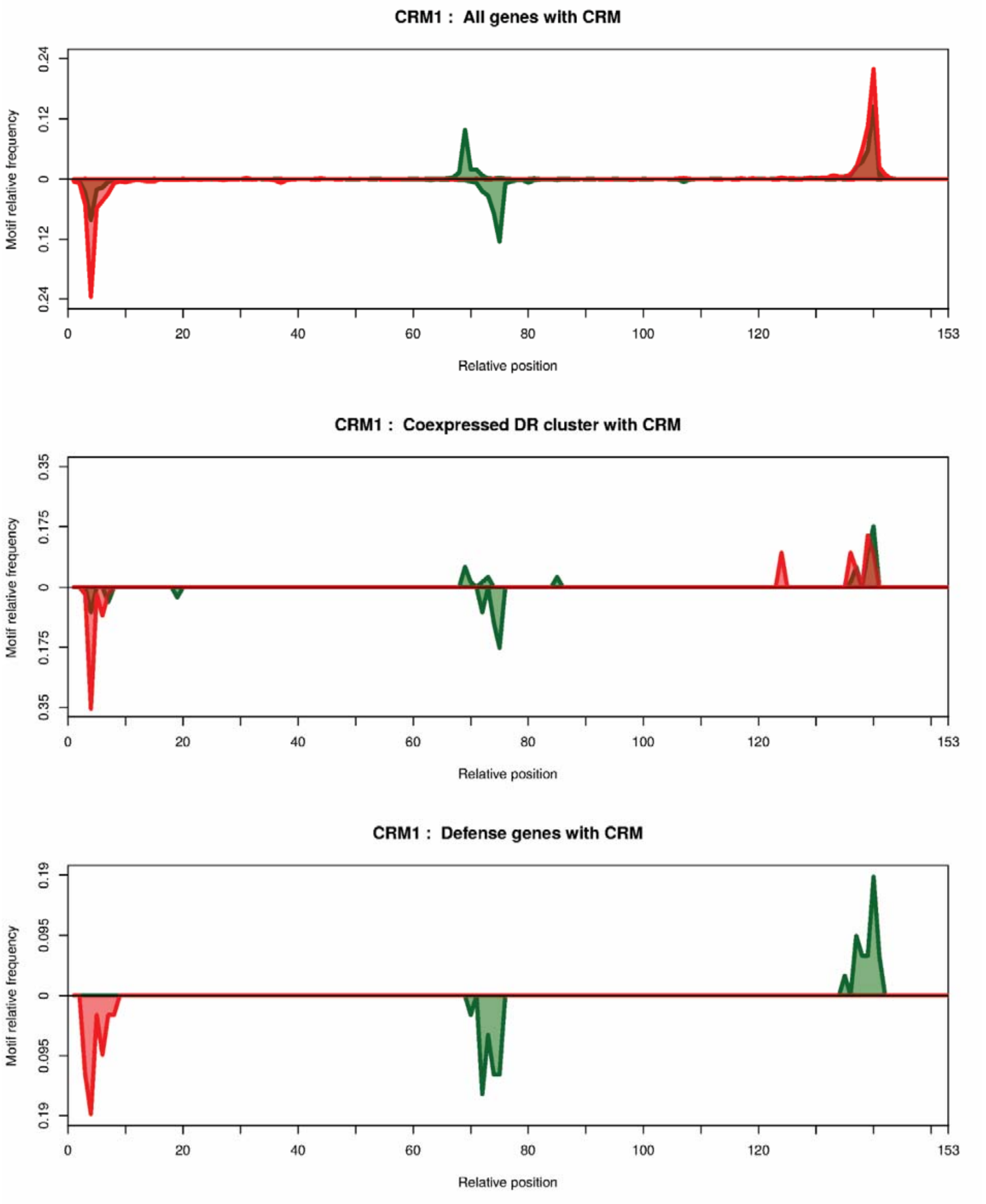

(A) CRM1

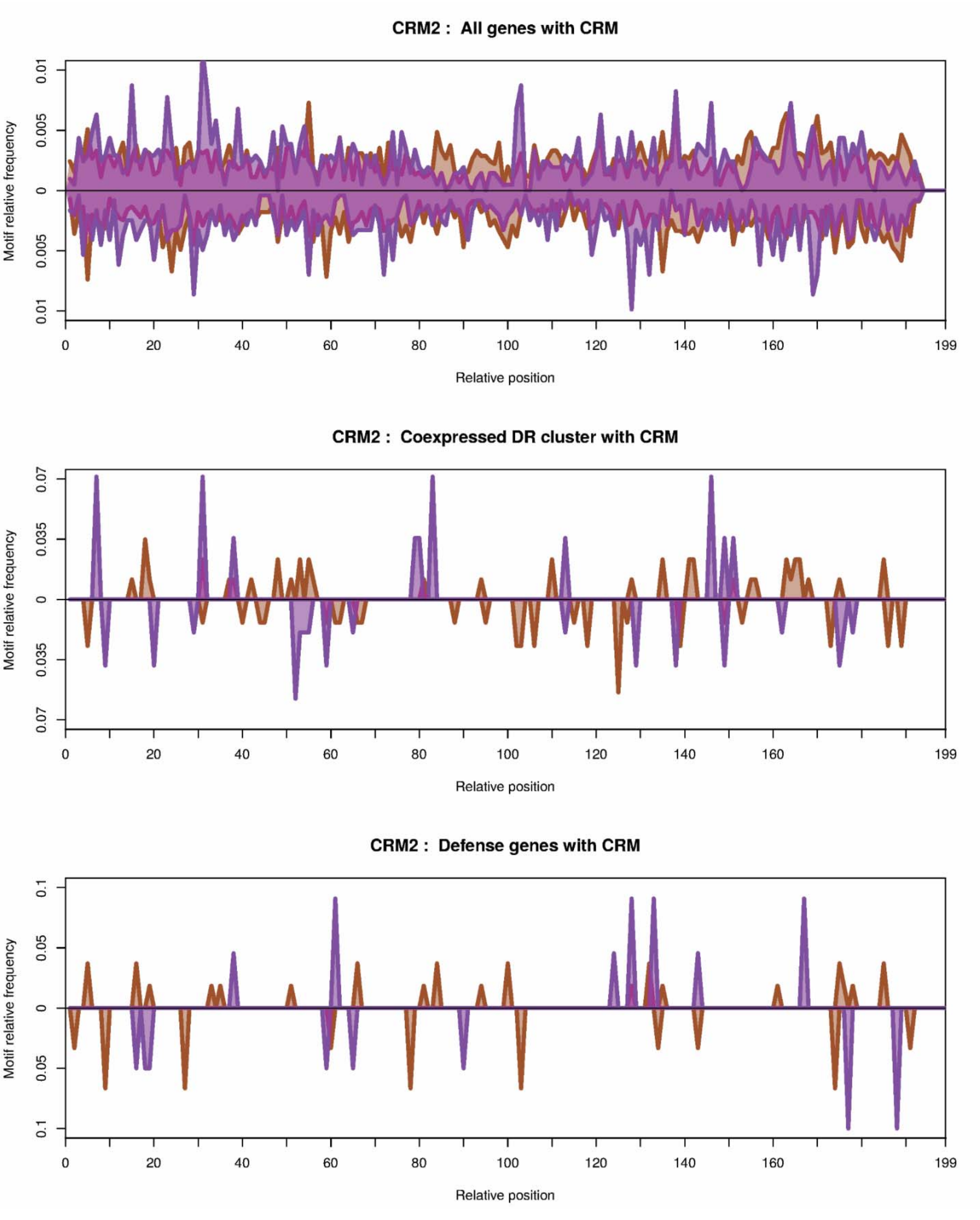
CRM2 : All genes with CRM
Motif relative frequency
0.01
0.005
0
0.005
0.01
0
20
40
60
80
100
120
140
160
199
Relative position
CRM2 : Coexpressed DR cluster with CRM
Motif relative frequency
0.07
0.035
0
0.035
0.07
0
20
40
60
80
100
120
140
160
199
Relative position
CRM2 : Defense genes with CRM
Motif relative frequency
0.1
0.05
0
0.05
0.1
0
20
40
60
80
100
120
140
160
199
Relative position

(B) CRM2

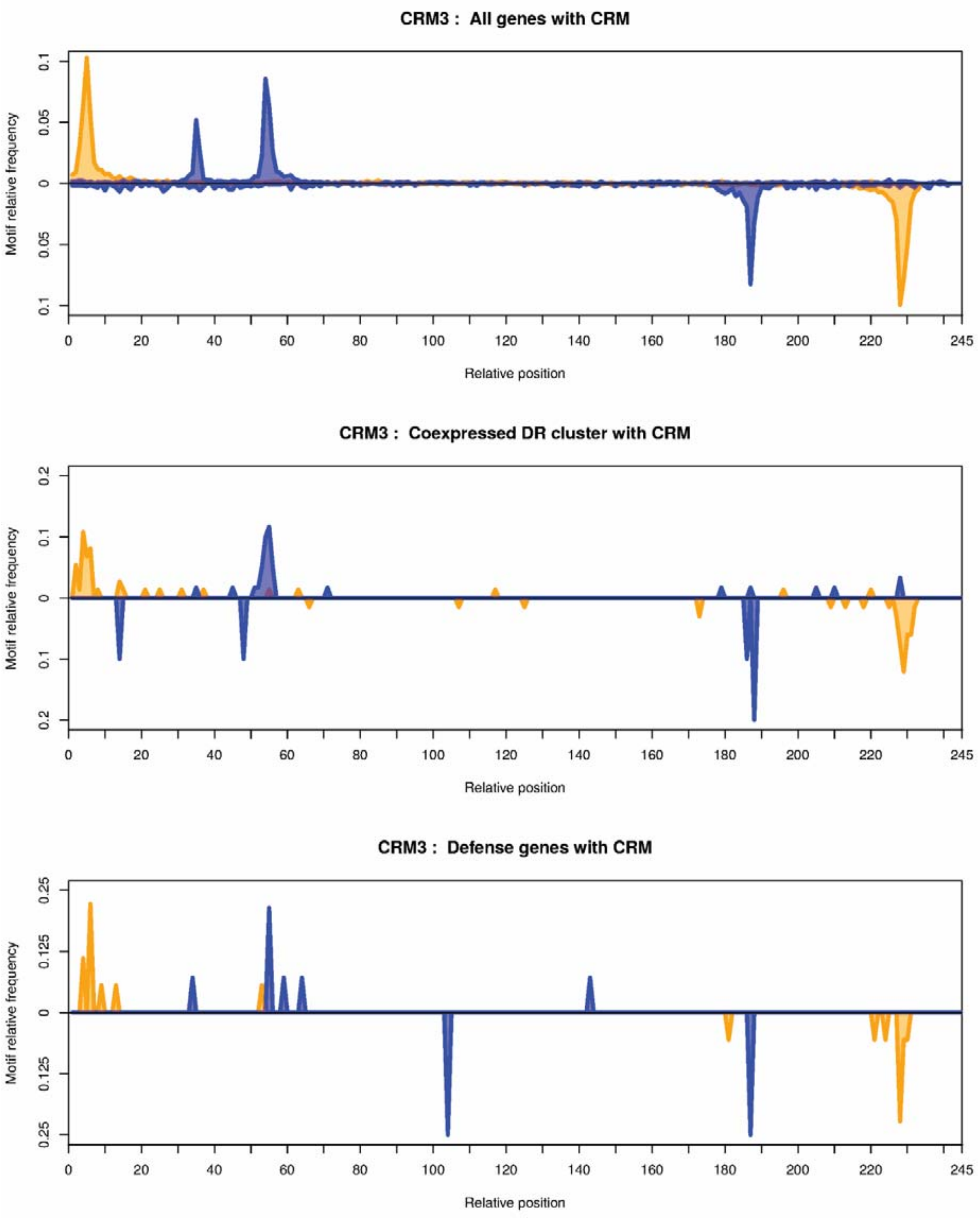
CRM3 : All genes with CRM
Motif relative frequency
0.1
0.05
0
0.05
0.1
0 20 40 60 80 100 120 140 160 180 200 220 245
Relative position
CRM3 : Coexpressed DR cluster with CRM
Motif relative frequency
0.2
0.1
0
0.1
0.2
0 20 40 60 80 100 120 140 160 180 200 220 245
Relative position
CRM3 : Defense genes with CRM
Motif relative frequency
0.25
0.125
0
0.125
0.25
0 20 40 60 80 100 120 140 160 180 200 220 245
Relative position

(C) CRM3

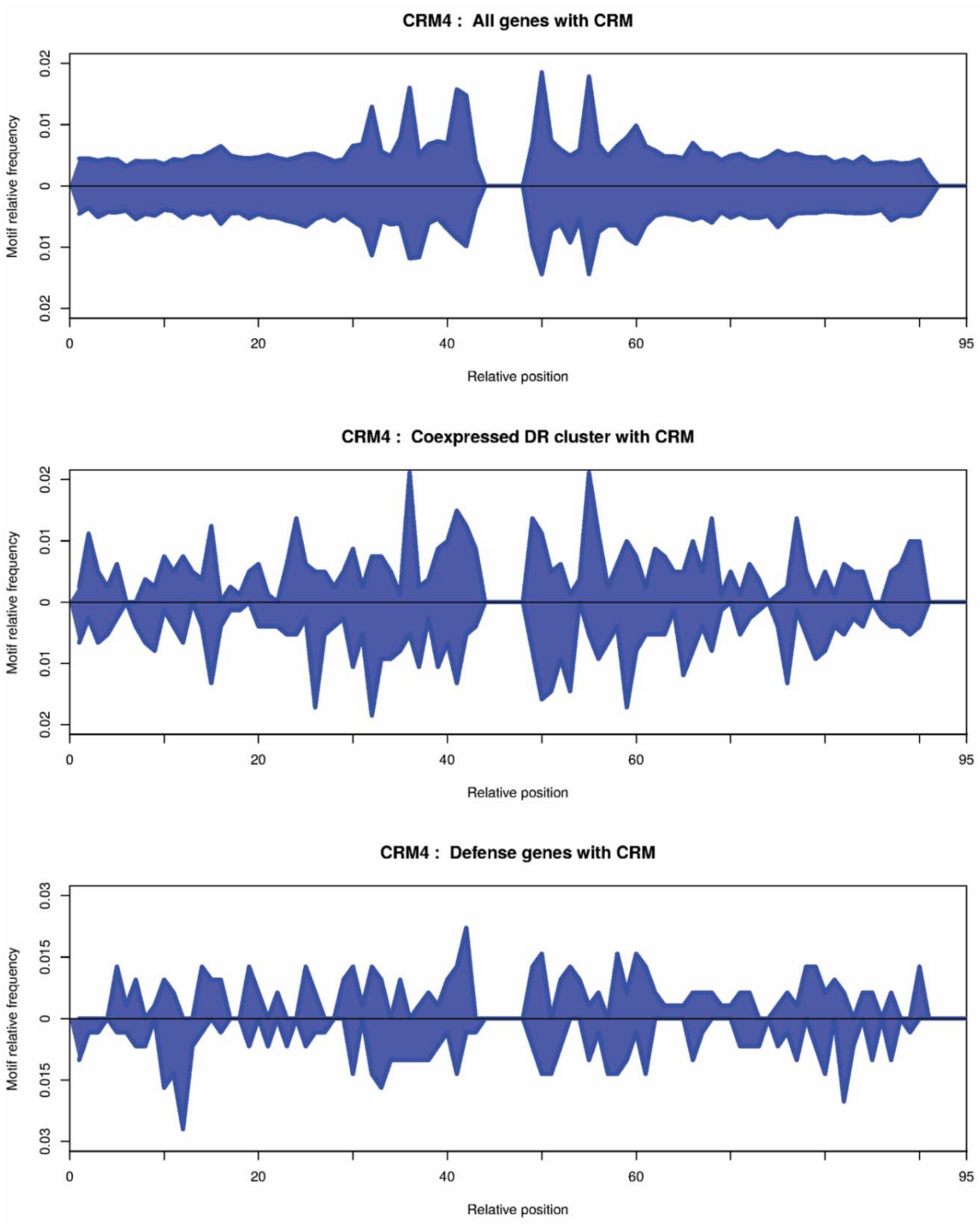

CRM4 : All genes with CRM
Motif relative frequency
Relative position
CRM4 : Coexpressed DR cluster with CRM
Motif relative frequency
Relative position
CRM4 : Defense genes with CRM
Motif relative frequency
Relative position

(D) CRM4

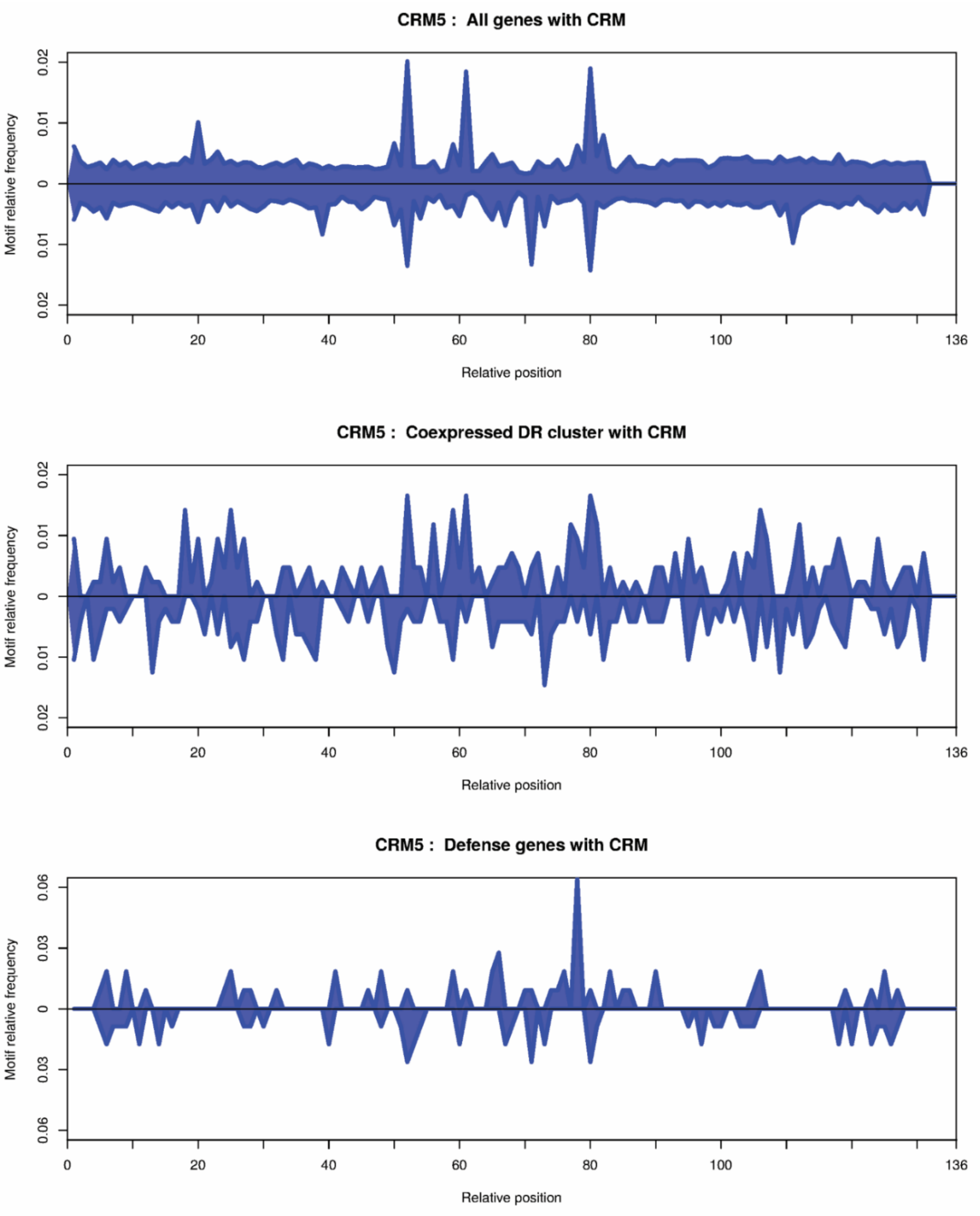

(E) CRM5

Figure S4. CRM motif densities. Frequency for each nucleotide position across the CRM1-5 (A-E) windows was calculated as a ratio of total occurrences of the constituent motif in the CRM found in all genes, BS-DR cluster genes, and FA-DR genes (“Defense Genes”). The strand which the motif is found is given as either below (- strand) or above (+ strand) the Y-axis origin.

Figure S5.

```
>CRM1
tTgaAGtATTAAaataaaattaaaAAaAAAAcaAATTaCAcAgtTtGcgtgTAAATTGCG
AGACGaATcTTTTAAGCCTAATTacTCCATgATTtGcaaAATGTGtTgCTacAGtAaaca
tTTGctAaTcATGacGGAtTAATTAGGCTTAA
```

65%

```
>CRM1-appended
tTgaAGtATTAAaataaaattaaaAAaAAAAcaAATTaCAcAgtTtGcgtgTAAATTGCG
AGACGaATcTTTTAAGCCTAATTacTCCATgATTtGcaaAATGTGtTgCTacAGtAaaca
tTTGctAaTcATGacGGAtTAATTAGGCTTAATgCgttATgAtTTgataatGTtGTgcTa
TgGtaATatcttaTTacTAaTgAtgGATTAAatAGatTtaAAAgATTCGTCTCGCAATTT
ACAcGCAAAtTgTGTAATTAGTTTTTTTATTAGTcTACgTTTAATACTTcATacATGTGT
ctAcaATATtTATGATGTGACAcgctaAAAAATTTT
```

67%

```
>CRM3
ACTCCCTCCGTCCCaaAAattTaaaaagGacatttttttgatTTTTttttacaAtGTTTGA
CCGTTCGTCTTATTtAAAAAAtTTTTgtatAATtatTAAaataaAattTttgTataacTT
AAAgTATtaTTaAtAaTatATcagttAaTaacAAtataaattttttataaTtTTtaaAAAa
atTTTTTaAATAAGACGAAcGGTCAAACaTtgtaaaaaaaAAaaAaatcaacgtTgtcat
aTATTtTGGGACGGAGGGAGT
```

52%

```
>CRM3 in OsGLP8-6
ACTACCTCCGTCACAGAATATAATAATCTAGGACTGGATTAGACATATCCTAGTCAAACA
AATCTAGACAGCCCTTATCCAGATTCATTCAAGGATATGTCCCATCCAGTCCCAAATTGC
TATATTTTGGGATGGAGGAAGT
```

—

Figure S5. Consensus sequences of CRM1 and CRM3. The consensus sequence shown is a visual representation of the percentage of aligned nucleotides across every occurrence of each respective CRM in FASTA format. Each letter is the majority nucleotide for each position on the alignment. Capital letters represent nucleotides that are conserved in at least 50% of all sequences, and lowercase letters are less than 50%. The percentage shown on the right is based on the ratio of capital letters to lowercase letters. The length of each sequence is: CRM1 – 152 nt, CRM1-appended – 336 nt, CRM3 – 234 nt. Two CRM1 consensus sequences are shown. The first is generated based on the CRM1 window as seen in Fig. 4. The second, "CRM1-appended," is generated from CRM1 occurrences with an additional 184 nt extended from the 3' end. This was due to the evidence of a larger "self-complementary" motif structure seen in FA-DR genes (Fig. S8A). The CRM3 occurrence in the *OsGLP8-6* promoter is illustrated for comparison.

Figure S6.

(A) CRM1-appended / miR6225

```
    Query: 198-336
    sbi-MIR6225: 164-304
    score: 326
    evalue: 6e-20

UserSeq          198  uaaugauggauuaaauagauuuaaaagauucgucucgcaauuuacacgcaaauuguguaauuaguuuuuuuauuagucuacguuuaauacuucauacauguguc--uacaauauuuaugaugugacacgcuaaaaaauuuu  336
                      |||| ||   |||| |||| |||||||||||| |||||| |||||  |||||| | ||||||||||| | ||  ||||  |||| |  || || |||||| |    | | |  |||||||| || |   | |||| ||||
sbi-MIR6225      304  uaauaauaauuuaauuagacuuaaaagauucguuucgcaaauuacagacaaauuauauaauuaguuuuuauuuucaucuauauuuaguguuuaaugcaugugccguaagauucauuaugaugggaaaucuugaaaaguuuu  164

    Query: 198-299
    sbi-MIR6225: 1-102
    score: 312
    evalue: 9e-19

UserSeq          198  uaaugauggauuaaauagauuuaaaagauucgucucgcaauuuacacgcaaauuguguaauuaguuuuuuuauuagucuacguuuaauacuucauacaugug  299
                      |||| ||| | ||| |||| | ||||||||| |||| | ||||| |   ||||||| |||||||||| | || |||||  |||||| |||||  ||||||
sbi-MIR6225        1  uaaucauguacuaacuagacucaaaagauucaucucccgauuuauagauaaauugugcaauuaguuuuuguuuuugucuauauuuaaugcuucacgcaugug  102

    Query: 2-94
    sbi-MIR6225: 2-94
    score: 258
    evalue: 3e-14

UserSeq            2  ugaaguauuaaaauaaaauuaaaaaaaaaacaaauuacacaguuugcguguaaauugcgagacgaaucuuuuaagccuaauuacuccaugauu  94
                      ||||| ||||||    | |  |||| |||||| |||| |||| |||    | ||||| | |||| |||||||| || ||| ||| | |||||||
sbi-MIR6225       94  ugaagcauuaaauauagacaaaaacaaaaacuaauugcacaauuuaucuauaaaucgggagaugaaucuuuugagucuaguuaguacaugauu  2

    Query: 2-94
    sbi-MIR6225: 211-303
    score: 249
    evalue: 1e-13

UserSeq            2  ugaaguauuaaaauaaaauuaaaaaaaaaacaaauuacacaguuugcguguaaauugcgagacgaaucuuuuaagccuaauuacuccaugauu  94
                      | ||  | ||||    | || |||| |||||| ||||| | | ||||  ||||| |||||| ||||||||||||| |||||||    || |||
sbi-MIR6225      211  uuaaacacuaaauauagaugaaaauaaaaacuaauuauauaauuugucuguaauuugcgaaacgaaucuuuuaagucuaauuaaauuauuauu  303
```

(B) CRM1-appended / Harb-MITE_0L024C_6443

```
>DTHm_#DNA/Harb-MITE_0L024C_6443
          Length = 356

 Score =  186 bits (94), Expect = 4e-46
 Identities = 123/132 (93%), Gaps = 4/132 (3%)
 Strand = Plus / Minus


Query: 22  aaaaaaaaaacaaattacacagtttgcgtgtaaattgcgagacgaatcttttaagcctaa 81
            |||||||||| |||||||||||||||||||||||||||||||||||||||||||||||||
Sbjct: 263 aaaaaaaaaaccaattacacagtttgcgtgtaaattgcgagacgaatcttttaagcctaa 204


Query: 82  ttactccatgatttgcaaaatgtgttgctacagtaaacatttgctaatcatgacggatta 141
            || | |||||||||| | |||||| |||||||||||||||||||||   |||||||||
Sbjct: 203 ttgcgccatgatttg-acaatgtggtgctacagtaaacatttgctaat---gacggatta 148


Query: 142 attaggcttaat 153
            ||||||||||||
Sbjct: 147 attaggcttaat 136


 Score =  119 bits (60), Expect = 8e-26
 Identities = 78/84 (92%)
 Strand = Plus / Plus


Query: 218 ttaaaagattcgtctcgcaatttacacgcaaattgtgtaattagtttttttattagtcta 277
            ||||||||||||||||||||||||||||||| ||||||||| |||||||| ||   ||||
Sbjct: 210 ttaaaagattcgtctcgcaatttacacgcaaactgtgtaattggtttttttttttttcta 269


Query: 278 cgtttaatacttcatacatgtgtc 301
            | ||||||||||||||||||||||
Sbjct: 270 catttaatacttcatacatgtgtc 293
```

(A) CRM1 alignment with miRNA and MITE sequences. Top alignment with miRNA stem loop to CRM1-appended sequence. CRM1-appended consensus sequence is aligned to miRNA sequences or MITE sequence, Tc-Harbinger.

(A) CRM3 top alignments with known miRNAs

Accession	ID	Query Start	Query Stop	Subject Start	Subject Stop	Strand	Score	E-value
MI0005233	osa-MIR812a	47	222	33	197	-	307	2.00E-18
MI0019807	osa-MIR812r	23	222	25	213	-	292	3.00E-17
MI0019800	osa-MIR812q	23	261	2	225	-	287	8.00E-17
MI0005237	osa-MIR812e	41	221	2	169	-	264	6.00E-15
MI0005234	osa-MIR812b	51	222	11	169	+	255	3.00E-14
MI0023432	osa-MIR818f	35	260	47	258	+	253	5.00E-14
MI0019807	osa-MIR812r	33	211	17	184	+	241	5.00E-13
MI0017255	osa-MIR812o	41	222	3	171	-	233	2.00E-12
MI0019817	osa-MIR812t	38	220	29	199	-	228	6.00E-12
MI0019800	osa-MIR812q	82	261	67	241	+	224	1.00E-11
MI0005251	osa-MIR818e	34	243	9	202	-	222	2.00E-11
MI0005233	osa-MIR812a	33	216	25	197	+	212	1.00E-10
MI0017246	osa-MIR812l	38	226	34	209	-	209	2.00E-10
MI0017245	osa-MIR812k	56	207	44	183	-	208	3.00E-10

(B) CRM3 / Tc-Mariner_Chr1_5045258-5045503_36894

```
          Length = 246

 Score = 75.8 bits (38), Expect = 8e-13
 Identities = 41/42 (97%)
 Strand = Plus / Minus

Query: 43  tttttttacaatgtttgaccgttcgtcttatttaaaaaattt 84
           |||||||| |||||||||||||||||||||||||||||||||
Sbjct: 210 tttttttaaaatgtttgaccgttcgtcttatttaaaaaattt 169


 Score = 75.8 bits (38), Expect = 8e-13
 Identities = 41/42 (97%)
 Strand = Plus / Plus

Query: 179 aaattttttaaataagacgaacggtcaaacattgtaaaaaaa 220
           ||||||||||||||||||||||||||||||||| ||||||||
Sbjct: 169 aaattttttaaataagacgaacggtcaaacattttaaaaaaa 210
```

(B) CRM3 alignment with miRNA and MITE sequences. A table of top alignments with miRNA stem loops is included due to the redundancy of matching results. CRM3 consensus sequences are aligned to miRNA sequences or MITE sequence, Tc-Mariner.

Figure S6. CRM1 and CRM3 consensus alignments with repeat elements. Top alignment results from CRM1-appended (A) and CRM3 (B) consensus sequences with miRNA stem loops from miRBase (www.mirbase.org) or repeat elements from RITE database (www.genome.arizona.edu/cgi-bin/rite/index.cgi). All alignment results are scored with E-values less than 1e-10.

Figure S7.

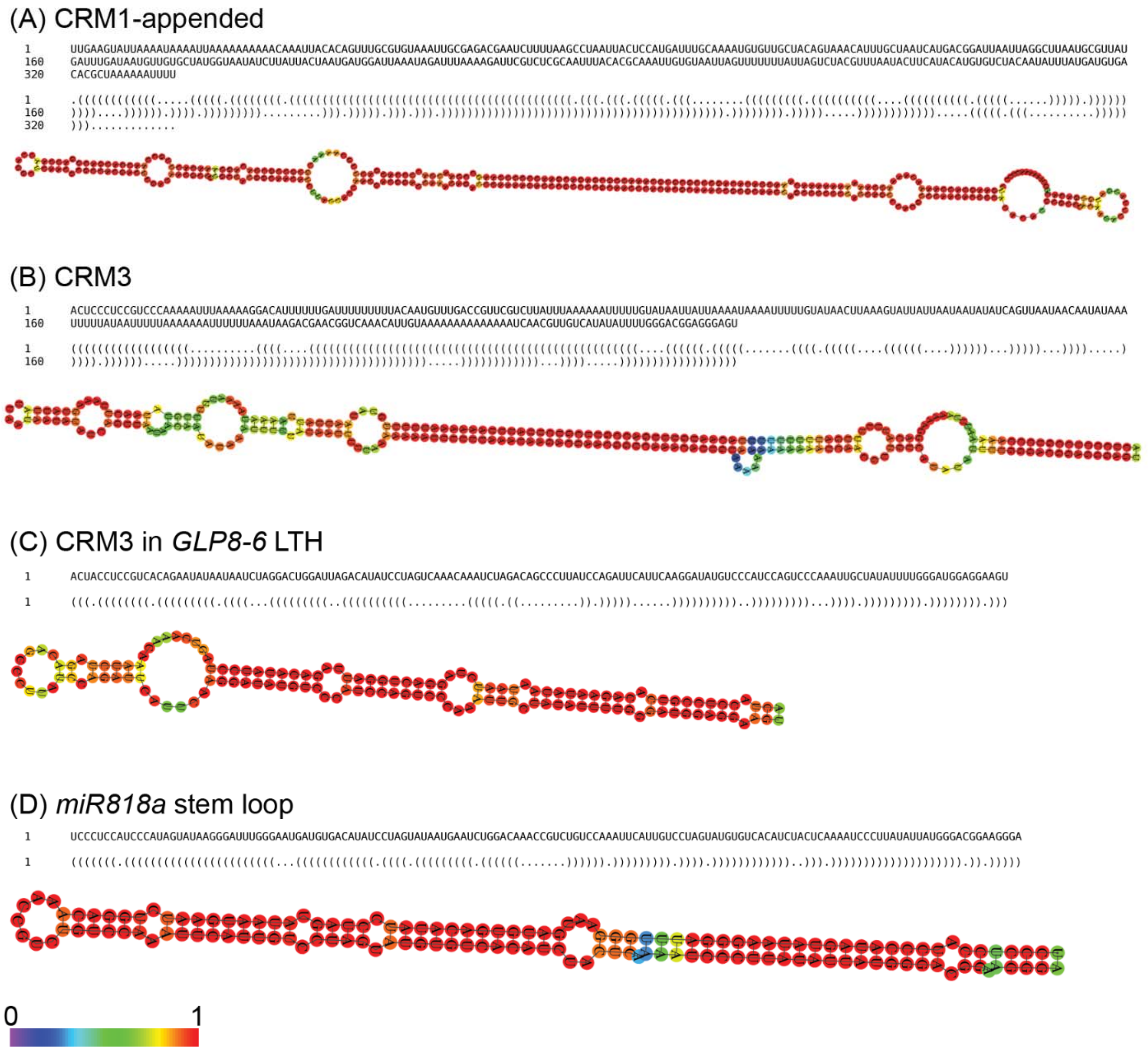

Figure S7. CRM1 and CRM3 secondary structure. Putative RNA secondary structure generated using the program RNAfold (rna.tbi.univie.ac.at/cgi-bin/RNAWebSuite/RNAfold.cgi). The optimal secondary structure based on minimum free energy is illustrated in dot-bracket notation and graphically using base pair probabilities.

Figure S8.

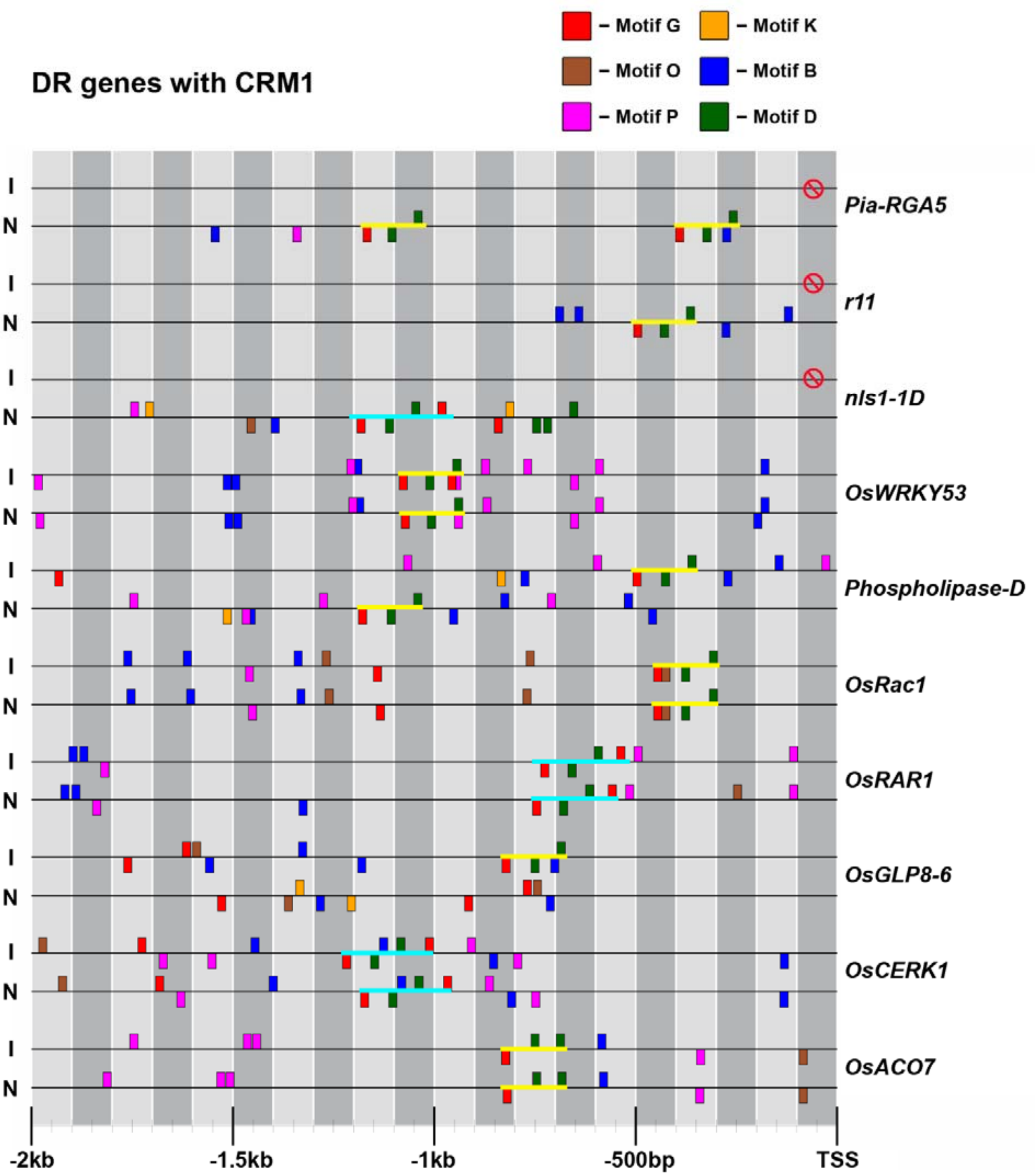

(A) CRM1 occurrences in DR genes.

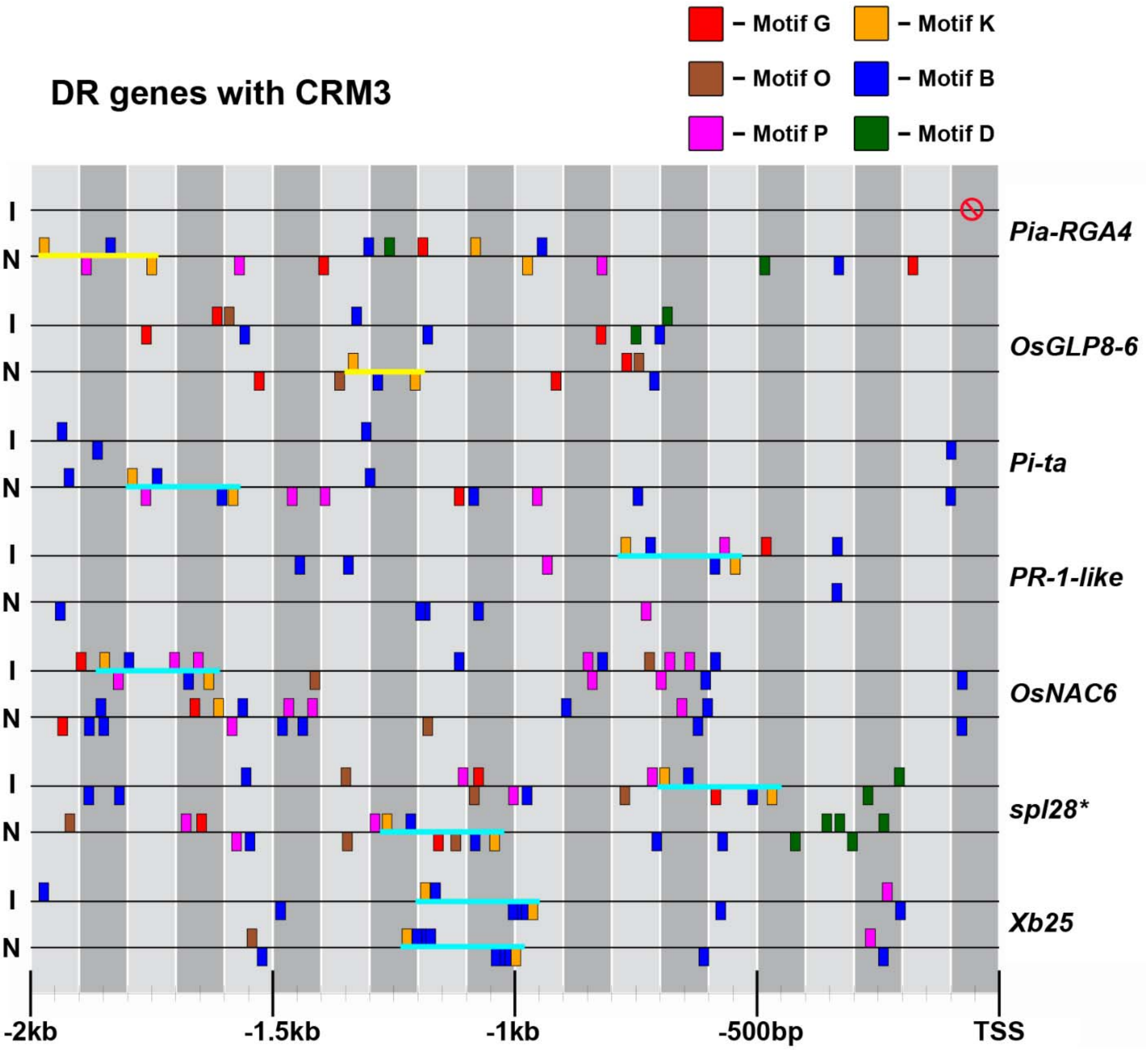

(B) CRM3 occurrences in DR genes.

Figure S8. CRM occurrences in known DR genes. The CRM motifs within each of the entire DR gene promoter for CRM1 (A) and CRM3 (B). Each DR gene promoter is from IR64 (I) or Nipponbare (N). Red interdictory circles indicate a lack of promoter and/or gene sequence in the variety. CRMs are highlighted in bright yellow or cyan. Cyan-highlighted regions are the larger, "self-complementary" version of the respective CRM. The cyan-highlighted versions of CRM1 are what prompted the generation of a "CRM1-appended" consensus sequence as seen in Figure S5. Cyan-highlighted CRM3 occurrences contain complementary Motif B sites.

Figure S9.

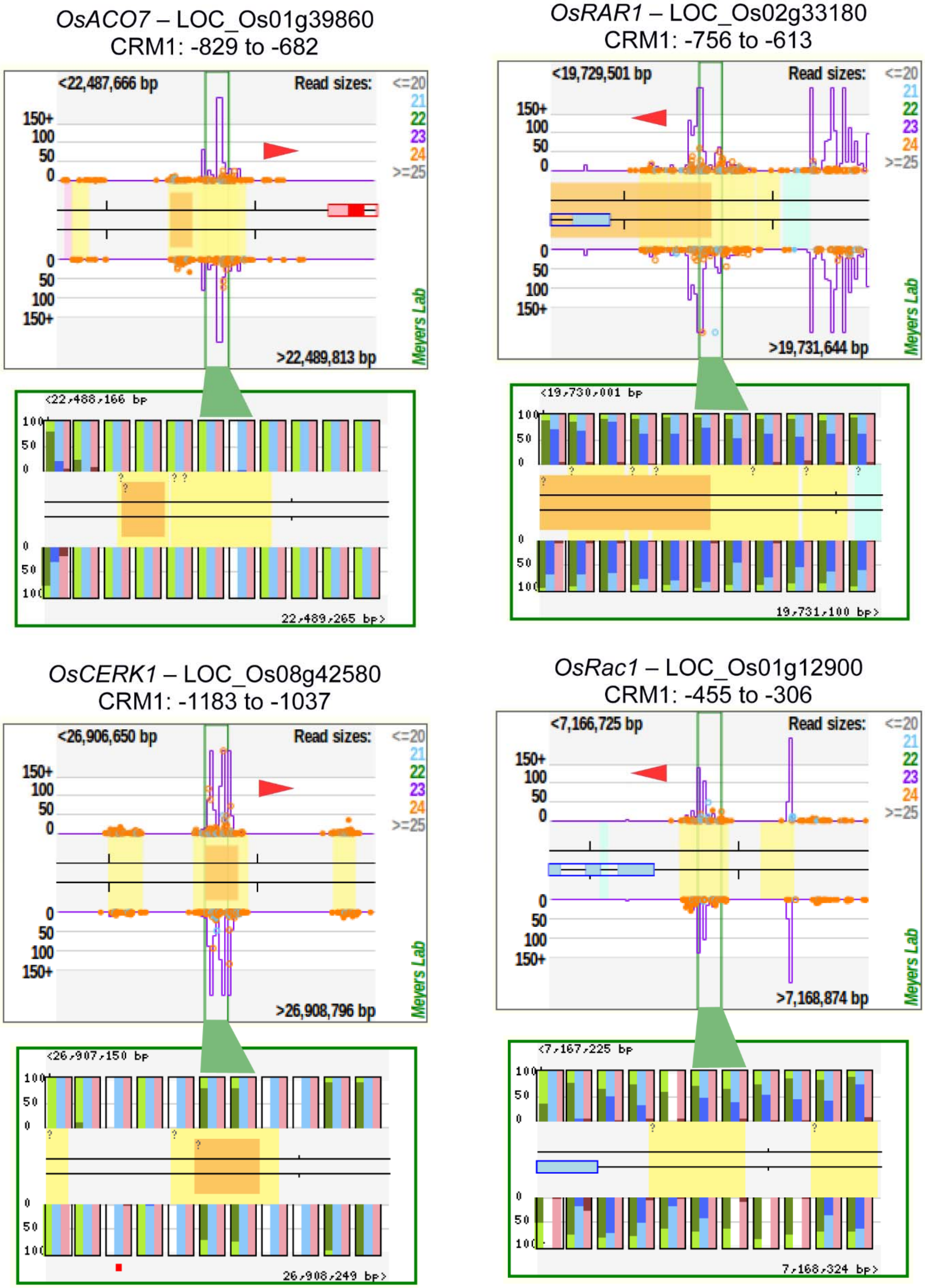
OsACO7 – LOC_Os01g39860
CRM1: -829 to -682
<22,487,666 bp
Read sizes:
<=20
21
22
23
24
>=25
150+
100
50
0
>22,489,813 bp
Meyers Lab
<22,488,166 bp
22,489,265 bp>
OsRAR1 – LOC_Os02g33180
CRM1: -756 to -613
<19,729,501 bp
>19,731,644 bp
<19,730,001 bp
19,731,100 bp>
OsCERK1 – LOC_Os08g42580
CRM1: -1183 to -1037
<26,906,650 bp
>26,908,796 bp
<26,907,150 bp
26,908,249 bp>
OsRac1 – LOC_Os01g12900
CRM1: -455 to -306
<7,166,725 bp
>7,168,874 bp
<7,167,225 bp
7,168,324 bp>

Phospholipase-D – LOC_Os01g07760
CRM1: -1188 to -1040

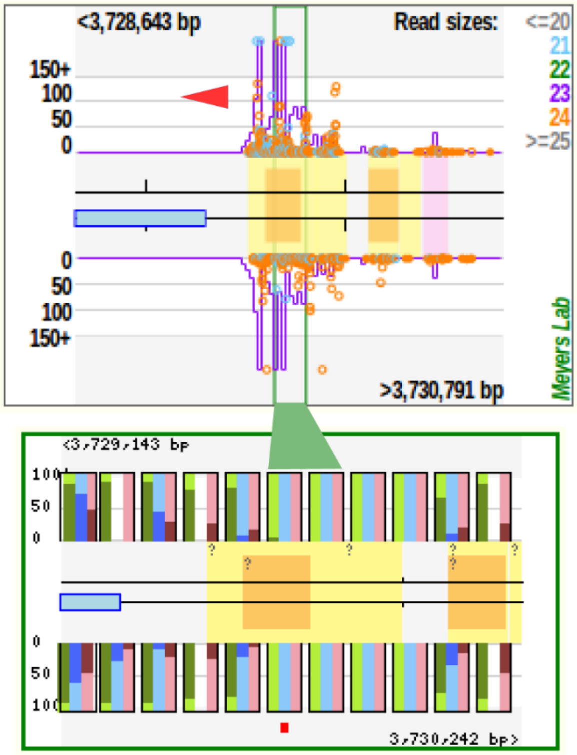

nls1-1D – LOC_Os11g14380
CRM1: -1192 to -1045

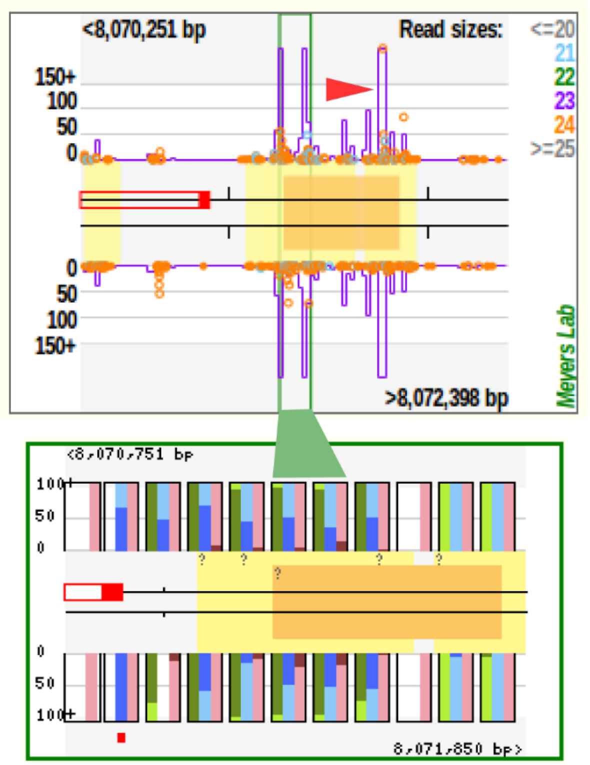

OsWRKY53 – LOC_Os05g27730
CRM1: -1082 to -941

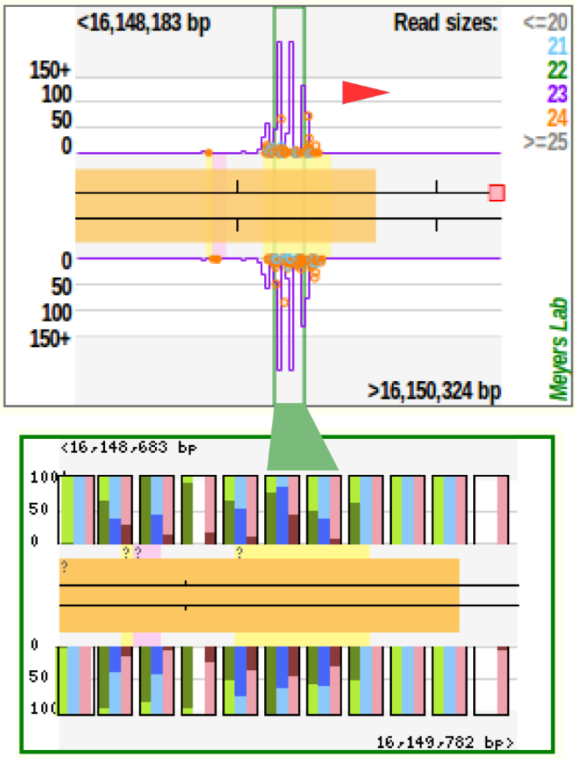

r11 – LOC_Os11g29920
CRM1: -505 to -363

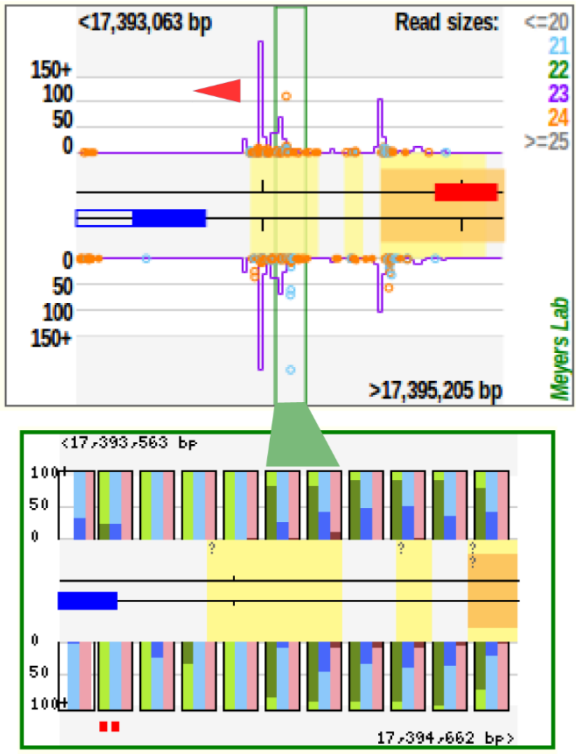

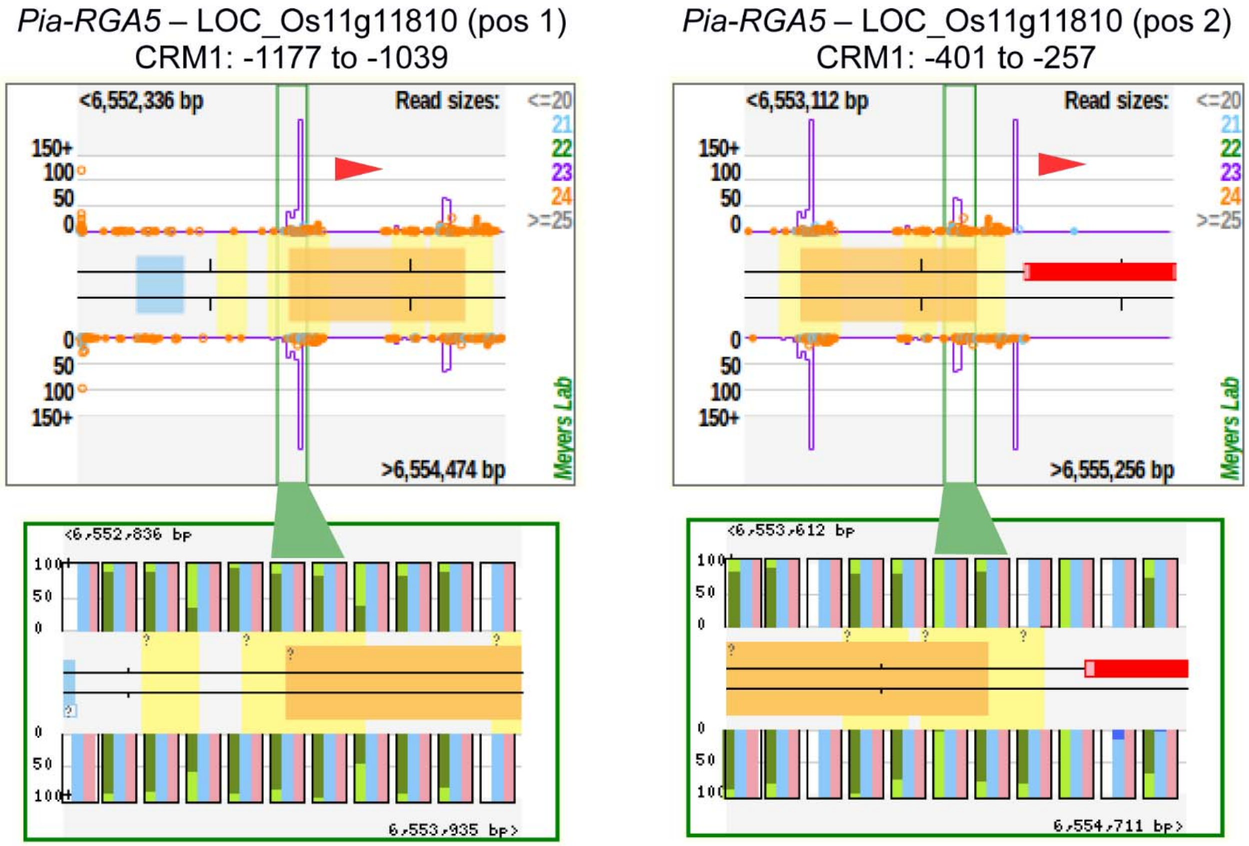

(A) CRM1 sRNA reads and methylation in known DR genes.

OsNAC6 – LOC_Os01g66120
CRM3: −1622 to -1350

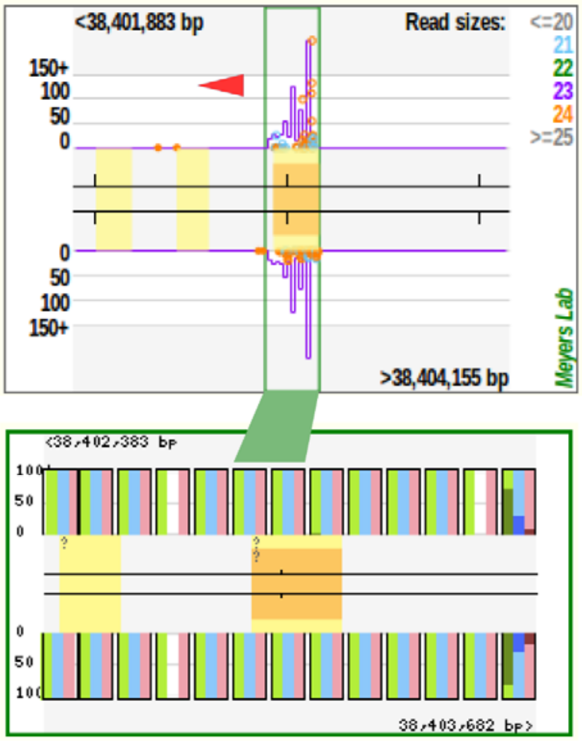

OsGLP8-6 – LOC_Os08g09000
CRM3: -1344 to -1203

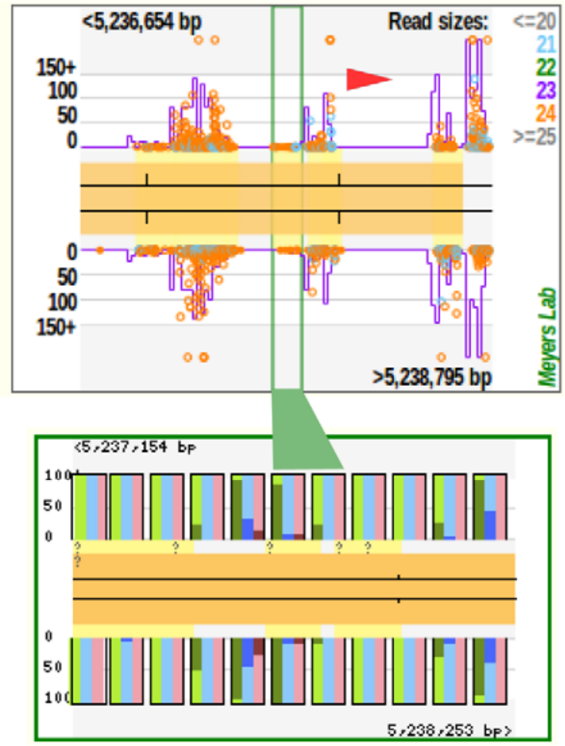

Pi-ta – LOC_Os12g18360
CRM3: -1800 to -1579

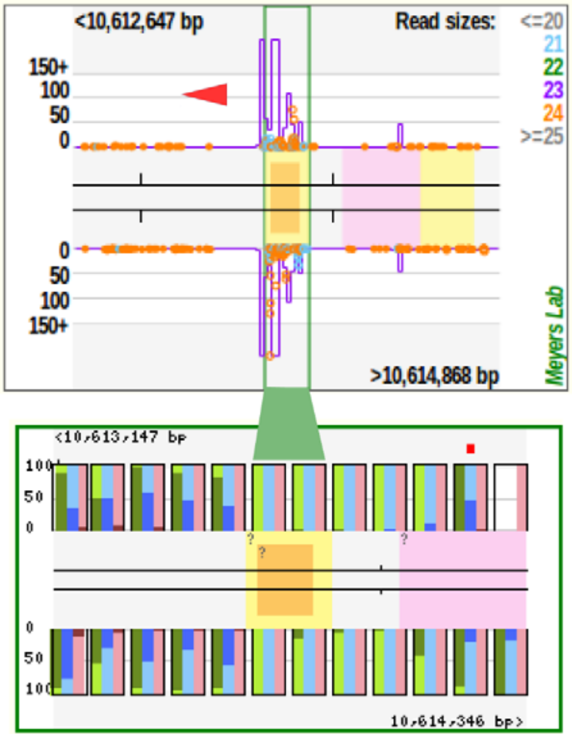

Pia-RGA4- LOC_Os11g11790
CRM3: -1982 to -1747

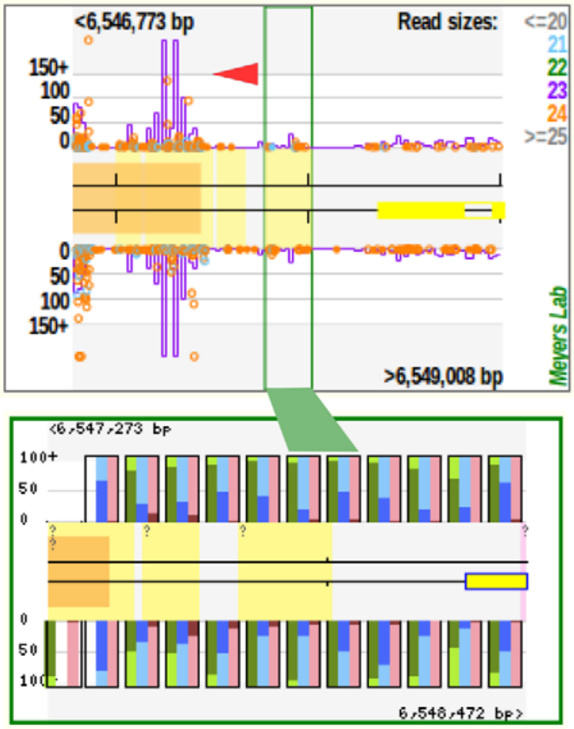

Xb25 – LOC_Os09g33810
CRM3: -1233 to -995

*spl28** - LOC_Os01g50770
CRM3: -1274 to -1039

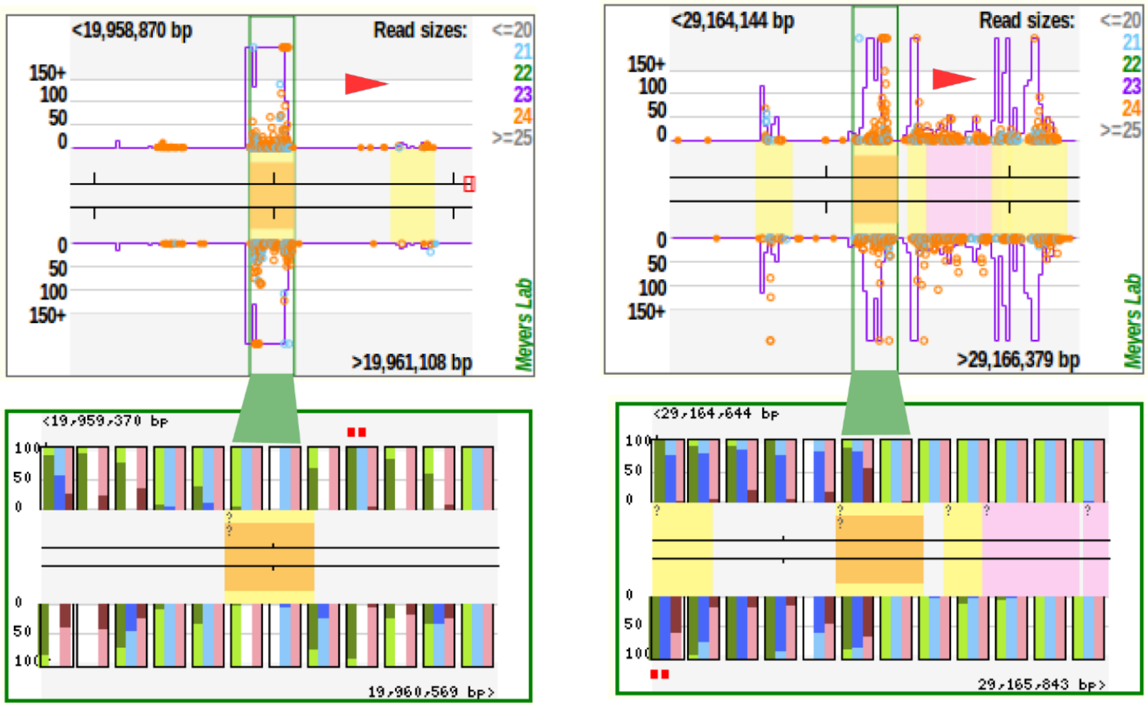

(B) CRM1 sRNA reads and methylation in known DR genes.

Figure S9. Total sRNA reads and methylation in CRM1 and CRM3 locations within known DR gene promoters. For each known DR gene containing a CRM1 (A) or CRM3 (B) a graphical representation of sRNA reads are shown, which was taken from the MPSS sRNA sequencing database (mpss.danforthcenter.org/dbs/index.php?SITE=rice_sRNA) (Nakano, 2006). Green boxes in the top plots (within a 2 Kb region) show the CRM position. Above and below the X-axis is plus or minus strand-aligned reads. The red arrow indicates the direction of the respective downstream DR gene. Orange dots are sRNA reads mapped to that location. The purple line graph shows the "K-mer" line, or degree of repetitiveness. A K-mer value of >50 indicates a lighly heterochromatic region, and known to produce sRNAs. The bottom graphs show total methylation within the 1kb surrounding the CRM location. Each partition along the X-axis represents a block of 100bp. Three separate methylations are represented: CG (green), CHG (blue), and CHH (red). Each colored bar is the relative percentage of methylated (darker color) to unmethylated (lighter color) reads. The green trapezoid from the top to bottom graphs shows where the CRM location resides in the methylation plot. [Nakano, M. *et al.* Plant MPSS databases: signature-based transcriptional resources for analyses of mRNA and small RNA. *Nucleic Acids Res* **34**, D731-735, doi:10.1093/nar/gkj077 (2006)].

Figure S10.

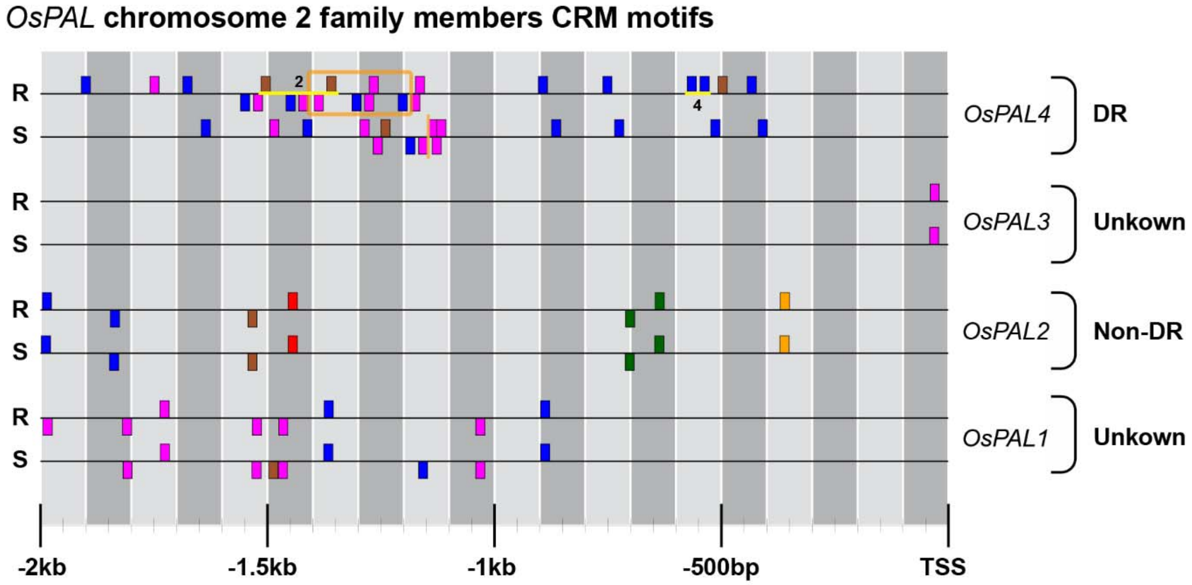

(A) Full OsPAL gene family CRM motif profiles. CRM motif profiles in resistant (IR64) and susceptible (Azucena) haplotypes.

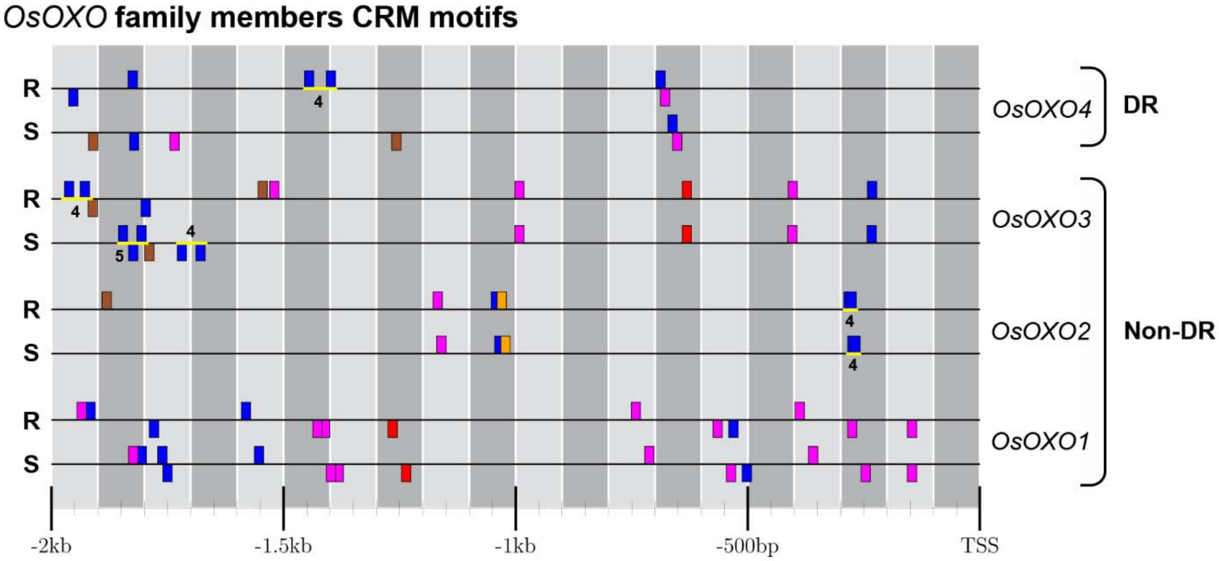

(B) Full *OsOXO* gene family CRM motif profiles. CRM motif profiles in susceptible (Vandana) haplotype. The resistant donor, Moroberekan, does not have available genome sequence, thus another japonica sub-population variety, Nipponbare, was used.

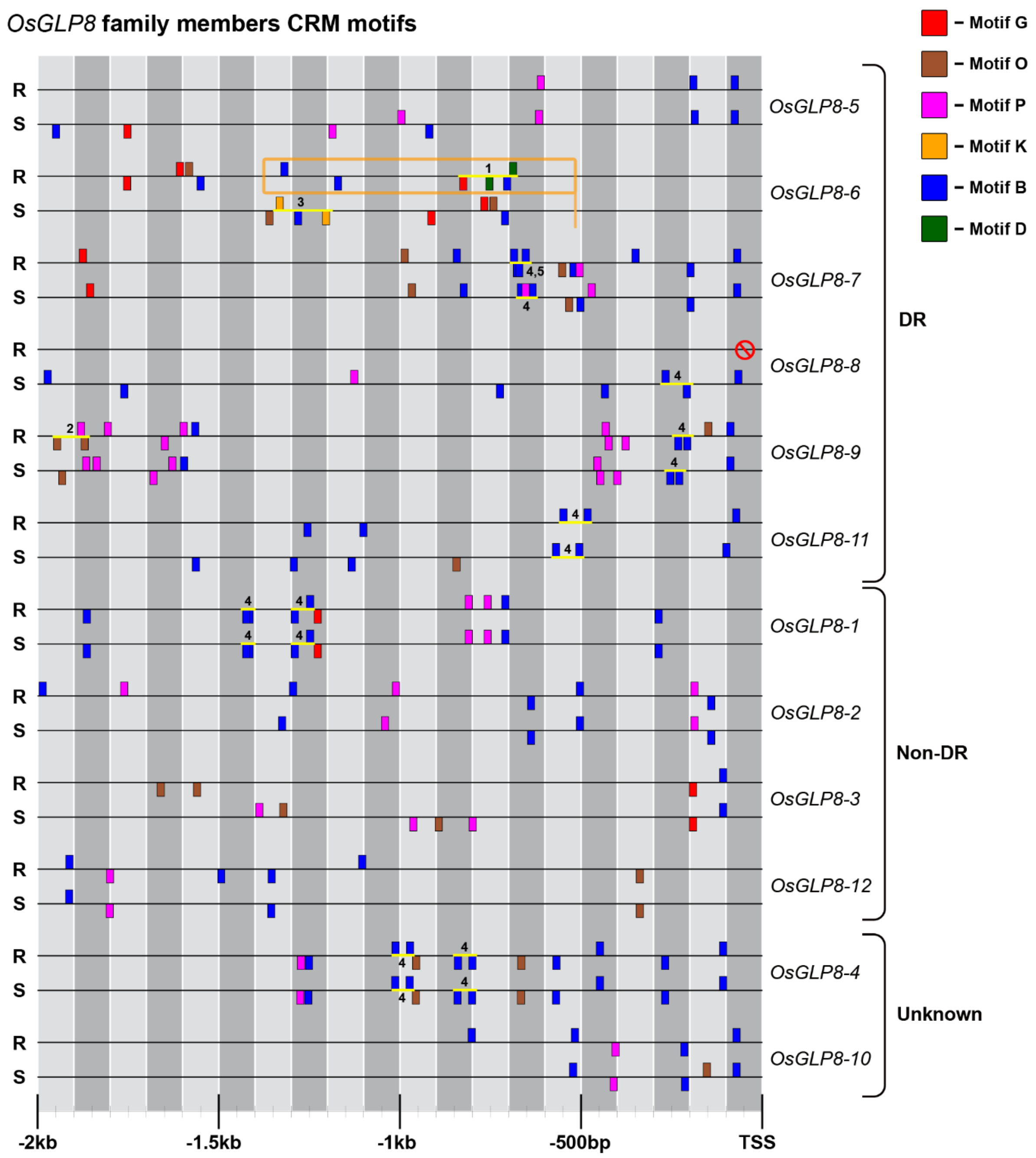

(C) Full OsGLP8 gene family CRM motif profiles. CRM motif profiles in resistant (SHZ-2) and susceptible (LTH) haplotypes.

Figure S10. Full gene family CRM motif profiles. The CRM motifs within each of the entire DR gene families are shown for *OsPAL* (A), *OsOXO* (B), and *OsGLP8* (C). These results include gene family members that have unknown functions in resistance positively or negatively. Red interdictory circles indicate a lack of promoter and/or gene sequence in the variety.

Supplementary information, TABLE LEGENDS

Table S1. Gene expression studies. Information on each experiment used in the defense response gene co-expression analysis. For each study, the treatment, rice variety used, tissue type, experimental conditions, expression data type (platform), and reference are given.

Table S2. Gene locus IDs within each co-expression cluster. The MSU7 annotation of each rice gene is given within the cluster identifier for each co-expressed group of genes found, totaling in 65 clusters.

Table S3. Co-expression cluster validation measures. Scores and statistics on the co-expression clusters found from each attempt at dendrogram branch cutting using different methods and parameters. Chosen parameter set: "H_MG=0.25".

Table S4. DR-related Gene Ontology terms. Gene Ontology identifiers from the Plant GOSlim database (rice.plantbiology.msu.edu/index.shtml) that are deemed as related to the plant defense response. These terms were used for testing for enrichment in co-expression clusters.

Table S5. Functionally-Associated DR (FA-DR) genes. A list of genes that are functionally validated to be involved in the rice DR to pathogens. Locus ID for the MSU7 Nipponbare reference genome is given. The disease in which the FA-DR gene is involved as well as the method of validation are shown. The effect, positive (+) or negative (-), is given, the reference for the gene, and annotation in the final columns.

Table S6. Co-expression clusters FA-DR gene enrichment tests. Each co-expressed gene cluster is labelled as a unique color name. The *P*-values given are a result of a Fisher Exact test based on the comparison of the number of FA-DR genes within the respective cluster or the rest of the genome. Only clusters with at least one FA-DR gene were tested. *P*-value correction was done using Benjamini-Hochberg (BH) procedure with a False Discovery Rate (FDR) of 0.05. Number of tests totaled to 36. One cluster, greenyellow, was found to be statistically enriched, and is named the "BS-DR" cluster for future analysis.

Table S7. Co-expression clusters DR-GO term enrichment tests. Each co-expressed gene cluster is labelled as a unique color name. The *P*-values given are a result of a Fisher Exact test on each DR-GO term, counting the genes labelled with the respective DR-GO term within each respective cluster or the rest of the genome. DR-GO terms were only tested on the clusters that contained at least one gene labelled with the respective term. *P*-value correction was done using Benjamini-Hochberg (BH) procedure with a False Discovery Rate (FDR) of 0.05. Number of tests totaled to 1476. The row colored red is the last ranked DR-GO term which is less than the BH-corrected value.

Table S8. Co-expressed gene clusters with enriched DR-GO terms. Twenty-eight total DR-GO terms are enriched, and are found across 17 clusters. Cluster "greenyellow" is the "BS-DR" cluster. Each cluster is given, followed by a list of DR-GO terms enriched in the respective cluster, along with the GO term annotation.

Table S9. Genes in BS-DR cluster. A list of the genes (including full annotations) found within the co-expressed BS-DR cluster. The MSU7 locus ID, representative gene model, chromosome and relative position, position on plus or minus strand are given. All genes are not transposable elements (is_TE = N). All are expressed and representative, meaning their transcripts have been validated, and the gene model is the model that encompasses all UTRs.

Table S10. CRM enrichment tests in all co-expression clusters. Genes with the respective CRM in their promoters were compared between genes within a co-expression cluster relative to the rest of the promoterome. Using a Fisher Exact test, the number of genes with a particular CRM (1-5) within their promoters was tested for enrichment within each co-expression cluster relative to the rest of the genome. Odds ratios and computed *P*-values are given as a result of the Fisher Exact test.

Table S11. sRNA reads at DR gene CRM loci in sRNA pathway experiments. Reads mapped to CRM1 and CRM3 loci in DR gene promoters is shown for both 21 and 24nt sizes. Two separate studies are examined, AGO immunoprecipitation (A) and sRNA processing mutants (B).
(A) AGO immunoprecipitation experiments. The total sRNA reads from Nipponbare tissue mapped to each CRM locus is in the first column. The following columns are sRNAs from immunoprecipitated AGO1 clade proteins (AGO1a, AGO1b, AGO1c), and AGO4 clade proteins (AGO4a, AGO4b, AGO16).
(B) sRNA processing mutants. Wild-type Nipponbare tissue total sRNA reads mapped to each locus is in the first column. Reads of sRNAs in three different processing mutants, *Dicer-Like 1* (*dcl1*), *Dicer-like 3* (*dcl3*), and *RNA-Dependent RNA-polymerase II* (*rdr2*).

Table S12. DR gene families *OsPAL*, *OsOXO*, and *OsGLP8* within QTL. The QTL found to encompass each DR gene family member is listed. QTL were taken from databases Q-TARO, GRAMENE ((archive.gramene.org/qtl/, qtaro.abr.affrc.go.jp) and recent literature (DOI given).
